# Ultramicropore-Confined Solvation and Interphase Regulation Unlock High-Performance Hard Carbon Anodes for Sodium-Ion Batteries

**DOI:** 10.1007/s40820-026-02300-x

**Published:** 2026-07-27

**Authors:** Shunyuan Tan, Zhiyuan Cheng, Jiahao Xing, Jingkai Gao, Zimo Huang, Hongshuai Hou, Zhongliang Tian, Yanqing Lai, Jie Li, Simin Li, Xiaobo Ji

**Affiliations:** 1 National Energy Metal Resources and New Materials Key Laboratory, School of Metallurgy and Environment, Central South University, Changsha, 410083 People’s Republic of China; 2 National Engineering Research Center of Low-Carbon Nonferrous Metallurgy, National Energy Metal Resources and New Materials Key Laboratory, Hunan Provincial Key Laboratory of Nonferrous Value-Added Metallurgy, Engineering Research Center of the Ministry of Education for Advanced Battery Materials, Changsha, 410083 People’s Republic of China; 3 College of Chemistry and Chemical Engineering, National Energy Metal Resources and New Materials Key Laboratory, State Key Laboratory of Powder Metallurgy, Central South University, Changsha, 410083 People’s Republic of China

**Keywords:** Sodium-ion batteries, Hard carbon, Ultramicropores, Nanoconfined desolvation, NaF-rich interphases

## Abstract

Hard carbon (HC) anodes are promising for sodium-ion batteries, yet achieving high initial Coulombic efficiency (ICE), large plateau contribution, and fast charge-transfer kinetics remains challenging due to insufficient control of micro–nanostructure and interphase chemistry. Here, we present a precursor-level molecular engineering strategy that simultaneously regulates sodium desolvation and interphase chemistry in HC. An iodine-mediated oxidative cross-linking process converts starch into spherical HC with uniformly distributed ultramicropores and carbonyl-rich surfaces. These nanoconfined pores are proposed to act as molecular sieves, preferentially excluding bulky solvent molecules while allowing PF_6_^−^-coordinated Na^+^ access, thereby favoring anion-enriched electrolyte structures under confinement. Surface carbonyls exhibit strong PF_6_^−^ affinity, which may promote fluorine-rich inorganic interphases on pore surfaces. The synergistic effects of anion-selective adsorption and confined desolvation are suggested to favor thin and robust NaF-rich interphases at external surfaces and within nanoconfined pore regions, contributing to reversible interfacial reactions and rapid Na^+^ storage kinetics. Consequently, the HC delivers an ICE of 88.4%, a reversible capacity of 352.9 mAh g^−1^ at 0.1C, excellent rate capability (288.9 mAh g^−1^ at 5C), and 95.6% capacity retention over 200 cycles. This work offers a molecular-level design paradigm integrating efficiency, capacity, and kinetics in HC anodes.

**Supplementary Information:**

The online version contains supplementary material available at 10.1007/s40820-026-02300-x.

## Highlights

- An iodine-mediated oxidative cross-linking strategy enables the synthesis of spherical hard carbon with uniformly distributed ultramicropores (< 0.9 nm) and carbonyl-rich surfaces from starch precursors.
- Ultramicropore-confined solvation favors anion-enriched electrolyte structures, while carbonyl groups promote NaF-rich inorganic interphases, enabling synergistic interfacial regulation.
- The optimized hard carbon delivers high initial Coulombic efficiency (88.4%), large plateau contribution (64.1%), excellent rate capability (288.9 mAh g^−1^ at 5C), and stable cycling (95.6% retention after 200 cycles)

**Supplementary Information:**

The online version contains supplementary material available at 10.1007/s40820-026-02300-x.

## Graphical abstract

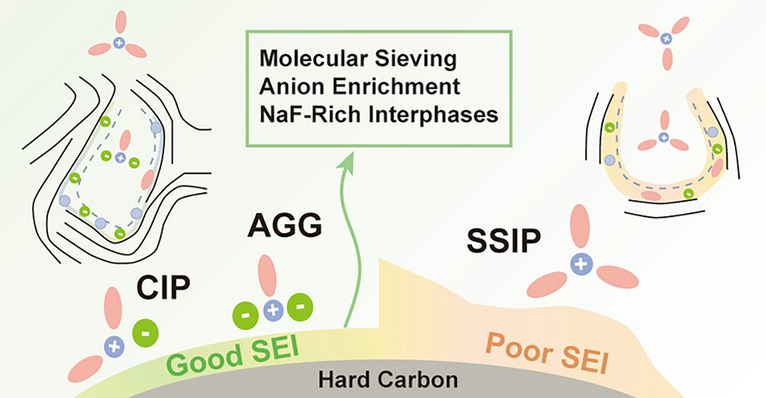

## Introduction

Sodium-ion batteries (SIBs) are emerging as promising complements to lithium-ion technologies for large-scale energy storage and electric mobility, driven by the natural abundance and low cost of sodium [1–4]. However, the larger ionic radius of Na^+^ (1.02 vs. 0.76 Å for Li^+^) leads to sluggish diffusion kinetics and pronounced volume variation during sodiation/desodiation [5, 6], which limiting the feasibility of conventional graphite and silicon–carbon anode materials in SIB systems. Hard carbon (HC) has therefore become the most viable anode material, owing to its disordered structure, enlarged interlayer spacing (> 0.37 nm), and nanoporous architecture, enabling relatively high capacity, low operating potential, and stable cycling. Nevertheless, However, sodium storage in HC involves multiple concurrent mechanisms, including surface adsorption, interlayer insertion, and pore filling, which complicate micro–nanostructural regulation and interfacial chemistry and often hinder the simultaneous achievement of high initial Coulombic efficiency (ICE), large plateau capacity, and fast charge-transfer kinetics [7, 8].

The electrochemical performance of HC is critically governed by its internal pore architecture and surface chemistry, both of which affect Na⁺ solvation, desolvation and interphase formation [9, 10]. Closed-pore size directly controls the thermodynamics of pore-confined sodium species that enlarged pores promote metallic Na clustering and shift storage towards deposition-like behavior [11], whereas ultramicropores (< 1 nm) stabilize Na clusters at higher potentials and favor plateau-region storage [12]. These pores also act as selective nanoreactors, enabling desolvation while restricting solvent ingress and thereby biasing solid electrolyte interphase (SEI) formation toward thin, inorganic-rich layers that lower interfacial resistance [13–16]. Surface functional groups further steer interphase chemistry. Some quinone-type C=O motifs strongly bind Na^+^ and catalyze excessive solvent decomposition, leading to thick and resistive SEI layers, whereas carbonyl C=O functionalities enable reversible Na⁺ adsorption and promote anion-derived inorganic SEI formation that facilitates fast ion transport [17, 18]. Collectively, these insights highlight that the synergistic regulation of ultramicropore confinement and surface-active functionalities is not merely beneficial but essential for unlocking high-efficiency, high-rate HC anodes.

From a materials-design perspective, the precursor chemistry, including molecular-chain configuration and functional-group distribution, critically governs the pore architecture and surface chemistry of HC. Considerable efforts have therefore focused on regulating the thermochemical evolution of carbon precursors through molecular engineering [19–21]. For example, acid-assisted pre-oxidation has been explored to regulate oxygen-functional-group evolution during carbonization [20]. Carbonyl-rich components exhibit high steric hindrance and strong conjugation with aromatic domains, which suppress oxygen adsorption and limit further oxidation reactivity. By contrast, pre-oxidatively introduced C(O)–O species can stabilize adjacent C–C bonds, thereby suppressing macromolecular depolymerization and inhibiting thermal rearrangement of carbon layers, ultimately promoting disordered frameworks with uniformly distributed closed micropores. Similarly, hydrogen-bond-mediated assembly enables molecular-level immobilization of glucose during dehydration and aromatization, yielding HC spheres with tunable crystallinity and enhanced electrochemical accessibility [21]. These studies collectively underscore the decisive role of precursor-level molecular interactions and functional-group chemistry in governing pore evolution and sodium-storage behavior in biomass-derived HCs.

Among various biomass precursors, starch is particularly attractive because of its high carbon content, natural abundance, and intrinsic spherical morphology, which favor dense particle packing and structural uniformity. However, direct pyrolysis of starch suffers from extensive glycosidic-bond cleavage, generating levoglucosan (LG) intermediates and volatile small molecules that induce severe structural collapse and carbon loss, often resulting in low carbon yield, excessive surface defects, and limited closed-pore formation [22, 23]. Although strategies such as esterification-induced cross-linking have been explored to stabilize the starch framework and preserve spherical morphology during pyrolysis [24, 25], many existing approaches still rely on complex processing, prolonged treatment, or limited structural controllability. Cost-effective and intrinsically controllable routes for simultaneously regulating pore evolution and surface chemistry therefore remain scarce.

Here we introduce a molecular-level cross-linking strategy that reconceptualizes starch-derived HC as a platform for the coordinated regulation of nanoconfined pore architecture and surface chemistry. By directing aromatization through iodine-mediated oxidative cross-linking, this approach stabilizes the spherical framework of starch-derived HC while promoting the formation of uniformly distributed ultramicropores (< 0.9 nm) and carbonyl-rich active sites. The resulting HC is proposed to facilitate nanoconfined desolvation and anion-enriched interfacial environments, which may contribute to improved interfacial charge transfer and sodium-storage reversibility. Consequently, the long-standing trade-off among ICE, plateau capacity, and rate capability can be effectively alleviated. This work establishes a general design principle in which precursor-level molecular engineering modulates solvation and interphase chemistry, offering a conceptual pathway towards high-performance HC anodes for next-generation SIBs.

## Experimental Section

### Reagents

Starch, iodine, and N-methyl-2-pyrrolidone (NMP) were purchased from Aladdin Biochemical Technology Co., Ltd. (China). The sodium-ion battery (SIB) electrolyte (1 M NaPF_6_ in EC/DEC = 1:1, vol%) was supplied by DoDochem Technology Co., Ltd. Sodium vanadium phosphate (NVP), polyvinylidene difluoride (PVDF), polytetrafluoroethylene (PTFE), carbon-coated aluminum foil, aluminum foil, Super P, and conductive carbon black (CB) were obtained from Shenzhen Kejing Star Technology Co., Ltd. All chemicals were used as received without further purification.

### Material Preparation

Starch (S) and iodine were first mechanically mixed at a mass ratio of 1:1 and pre-oxidized in a tubular furnace at 205 °C for 6 h under an Ar/O_2_ (8:2, v/v) atmosphere with a heating rate of 2 min^−1^, yielding a black powder denoted as IOS. For comparison, S was subjected to the same pre-oxidation treatment without iodine, producing a yellow powder labeled OS. The S, OS, and IOS precursors were then pyrolyzed at 600 °C for 1 h under flowing Ar at 5 °C min^−1^ to obtain S-600, OS-600, and IOS-600, respectively. Due to the severe foaming observed during this stage, S-600 and OS-600 were ball-milled into fine powders before further treatment. Finally, all intermediate products were carbonized at 1200 °C for 2 h under Ar with the same heating rate to yield the corresponding HCs, designated S-1200, OS-1200, and IOS-1200.

Notably, iodine-containing volatile species generated during oxidation, pyrolysis, and carbonization processes can, in principle, be captured and reintroduced into the precursor oxidation step, suggesting a potential closed-loop process design from an engineering perspective.

### Material Characterization

Thermogravimetric analysis (TGA550, USA) was performed to investigate the different pyrolysis behaviors of the precursors. Fourier transform infrared spectroscopy and mass spectrometry (TG-FTIR-MS, PerkinElmer TGA8000-FTIR-GCMS-ATD) was employed to analyze the gaseous products generated during the pyrolysis process. Fourier transform infrared spectroscopy (FTIR) spectra were collected to examine the evolution of functional groups (Thermo Fisher Scientific Nicolet iS20). X-ray diffraction (XRD, Rigaku SmartLab SE, Cu Kα radiation, λ = 0.154 nm) and Raman spectroscopy (Horiba LabRAM HR Evolution, 532 nm laser excitation) were used to characterize the crystalline structure of the HC. X-ray photoelectron spectroscopy (XPS) was conducted to determine the elemental composition and chemical states using a Thermo Scientific ESCALAB Xi + . The microstructural features were examined by scanning electron microscopy (SEM, TESCAN Vega3) and high-resolution transmission electron microscopy (HRTEM, JEOL F200). The total specific surface area (SSA) and pore-size distribution were determined by nitrogen adsorption–desorption measurements using a Quantachrome Autosorb IQ analyzer. Closed pores were further characterized by small-angle X-ray scattering (SAXS, Xenocs Xeuss 2.0).

### Electrochemical Measurement

The HC, Super P, and PVDF were mixed in a weight ratio of 90:2:8 in NMP to form a homogeneous slurry, which was subsequently coated onto carbon-coated aluminum foil. The prepared electrodes were dried in a vacuum oven at 80 °C for 12 h. The mass loading of the active material (electrode diameter: 10 mm) was approximately 2.0 mg cm^−2^. CR2032 coin cells were assembled to evaluate the electrochemical performance of the HC anode. Sodium metal foil was employed as both the counter and reference electrode, while 1 M NaPF_6_ dissolved in EC/DEC (1:1, vol%) was used as the electrolyte. Glass fiber (Whatman GF/F) served as the separator.

Galvanostatic charge/discharge (GCD) measurements were conducted using a LAND battery testing system within a voltage range of 0–2.0 V. Cyclic voltammetry (CV) tests were carried out at scan rates ranging from 0.1 to 1.0 mV s^−1^. The CV curves collected at different scan rates were used to analyze the sodium-ion storage kinetics according to the following relationship:

1 $$i=a{v}^{b}$$

2 $$lo\mathrm{g} i=b\mathrm{log}v+\mathrm{log}a$$

where $i$ represents the current response, $v$ refers to the scan rate, and $a$ and $b$ are variable parameters. The $b$ value is commonly used to evaluate the charge-storage kinetics. A *b*-value close to 0.5 suggests a diffusion-controlled process, whereas a value approaching 1.0 indicates a predominantly pseudocapacitive behavior.

In situ electrochemical impedance spectroscopy (EIS) measurements were performed using a Solartron Analytical 1470E eight-channel electrochemical workstation over a frequency range from 0.01 Hz to 100 kHz at a current density of 0.1C. The galvanostatic intermittent titration technique (GITT) was carried out on the LAND battery testing system with a pulse current of 0.05C applied for 1 h, followed by a relaxation period of 2 h. The sodium-ion diffusion coefficient (${D}_{{\mathrm{N}\mathrm{a}}^{+}}$) was calculated according to Fick’s second law using the following simplified equation:

3 $$\begin{array}{c}{D}_{{\mathrm{N}\mathrm{a}}^{+}}=\frac{4{\left(\frac{{m}_{\mathrm{b}}{V}_{\mathrm{m}}}{{M}_{\mathrm{b}}S}\right)}^{2}{\left(\frac{\Delta {E}_{\mathrm{s}}}{\Delta {E}_{\uptau }}\right)}^{2}}{\pi \tau }\end{array}$$

where $\tau (\mathrm{s})$, ${m}_{\mathrm{b}} (\mathrm{g})$, ${V}_{\mathrm{m}} (\mathrm{m}\mathrm{l} {\mathrm{m}\mathrm{o}\mathrm{l}}^{-1}$), and ${M}_{\mathrm{b}} (\mathrm{g} {\mathrm{m}\mathrm{o}\mathrm{l}}^{-1})$ are the pulse duration, the mass of the active material, the molar volume of the active material, and the molar mass of the active material, sequentially.

To further evaluate the practical sodium-storage performance, NVP//NPCS full cells were assembled in CR2032 coin cells using IOS-1200 as the anode and NVP as the cathode. The NVP cathode slurry was prepared by mixing 80 wt% NVP, 10 wt% conductive carbon black (CB), and 10 wt% PVDF in NMP solvent. The slurry was coated onto Al foil and dried in a vacuum oven at 100 °C for 24 h. The mass loading of the NVP cathode was approximately 1.1–1.2 mg cm^−2^. The activated IOS-1200 anode obtained from half-cells was subsequently paired with the NVP cathode to assemble the full cells using the same electrolyte. The N/P capacity ratio was controlled at approximately 1.2, and the full-cell measurements were conducted within a voltage range of 1.8–3.8 V.

For in situ XRD measurements, HC and PTFE were mixed in a weight ratio of 9:1 and rolled into a dry electrode as the anode. The electrode was assembled into a customized electrochemical cell in an argon-filled glovebox using a beryllium (Be) window as the X-ray transparent window. In situ XRD patterns were recorded on a Bruker D8 Advance diffractometer at room temperature under a constant current density of 0.1C over a diffraction angle range of 15°–35°.

For in situ Raman measurements, the electrochemical cell was assembled following a similar procedure to that used for in situ XRD, except that a quartz plate was employed as the optical window. Raman spectra were collected on a RENISHAW spectrometer using a 532 nm laser as the excitation source.

Small-angle X-ray scattering (SAXS) measurements were conducted to investigate the pore structure characteristics of the carbon samples. A Porod analysis method proposed by Stevens and Dahn was employed to analyze the SAXS data using the following simplified equation:

4 $$\begin{array}{c}I\left(Q\right)=\frac{A}{{q}^{a}}+\frac{{B}_{1}{a}_{1}^{4}}{{\left(1+{a}_{1}^{2}{Q}^{2}\right)}^{2}}+D\end{array}$$

where $I\left(Q\right)$ represents the scattering intensity as a function of the scattering vector $Q$. $A$ is the scaling factor associated with surface scattering in the low-$Q$ region, while ${B}_{1}$ corresponds to the pore-scattering contribution and is proportional to the total pore surface area. $D$ denotes the constant background term, and ${a}_{1}$ is the characteristic length related to the radius of spherical pores according to $R={a}_{1}\sqrt{10}$.

### Theoretical Calculation

Density functional theory (DFT) calculations were performed using the projector augmented wave (PAW) method as implemented in the Vienna ab initio simulation package (VASP) [26]. The generalized gradient approximation (GGA) with the Perdew–Burke–Ernzerhof (PBE) functional was employed to describe exchange–correlation interactions [27]. A plane-wave kinetic energy cutoff of 400 eV was used for structural optimizations [28]. The Brillouin zone was sampled using Monkhorst–Pack *k*-point grids [29]. Structural relaxations were carried out until the residual forces on each atom were less than 0.02 eV Å^−1^ and the total energy convergence threshold was set to 10⁻^5^ eV [28, 30]. Long-range dispersion interactions were accounted for using the zero-damping DFT-D3 method of Grimme [31].

To simulate closed ultramicropores in HC, carbon nanotube (CNT) models with different diameters were constructed as simplified structural descriptors. Although real HCs possess highly disordered structures with curved graphene layers, defects, and heteroatom functionalities, CNT-based models have been widely used to qualitatively evaluate pore confinement effects and Na adsorption behaviors in nanoporous carbons [13, 14, 32]. Therefore, the present calculations mainly aim to reveal the relative influence of pore size on Na adsorption behavior.

## Results and Discussion

### Molecular Engineering Strategy and Structural Evolution Mechanisms

Starch (S) is a widely used industrial product and an attractive precursor for HC synthesis owing to its low cost and environmental benignity. However, its direct pyrolytic conversion is intrinsically problematic, as thermal cleavage of glycosidic bonds generates volatile intermediates that induce severe foaming and structural collapse, as reflected by the disordered products S-600 and S-1200 obtained at different carbonization temperatures (Fig. S1a, d). Although thermal pre-oxidation can partially stabilize the polymeric framework, short oxidation times provide only limited cross-linking, so that pre-oxidized S (OS, 6 h) still undergoes pronounced foaming during subsequent pyrolysis at 600 °C (OS-600), while further carbonization at 1200 °C yields the corresponding HC, denoted as OS-1200 (Fig. S1b, e).

**Fig. 1:**
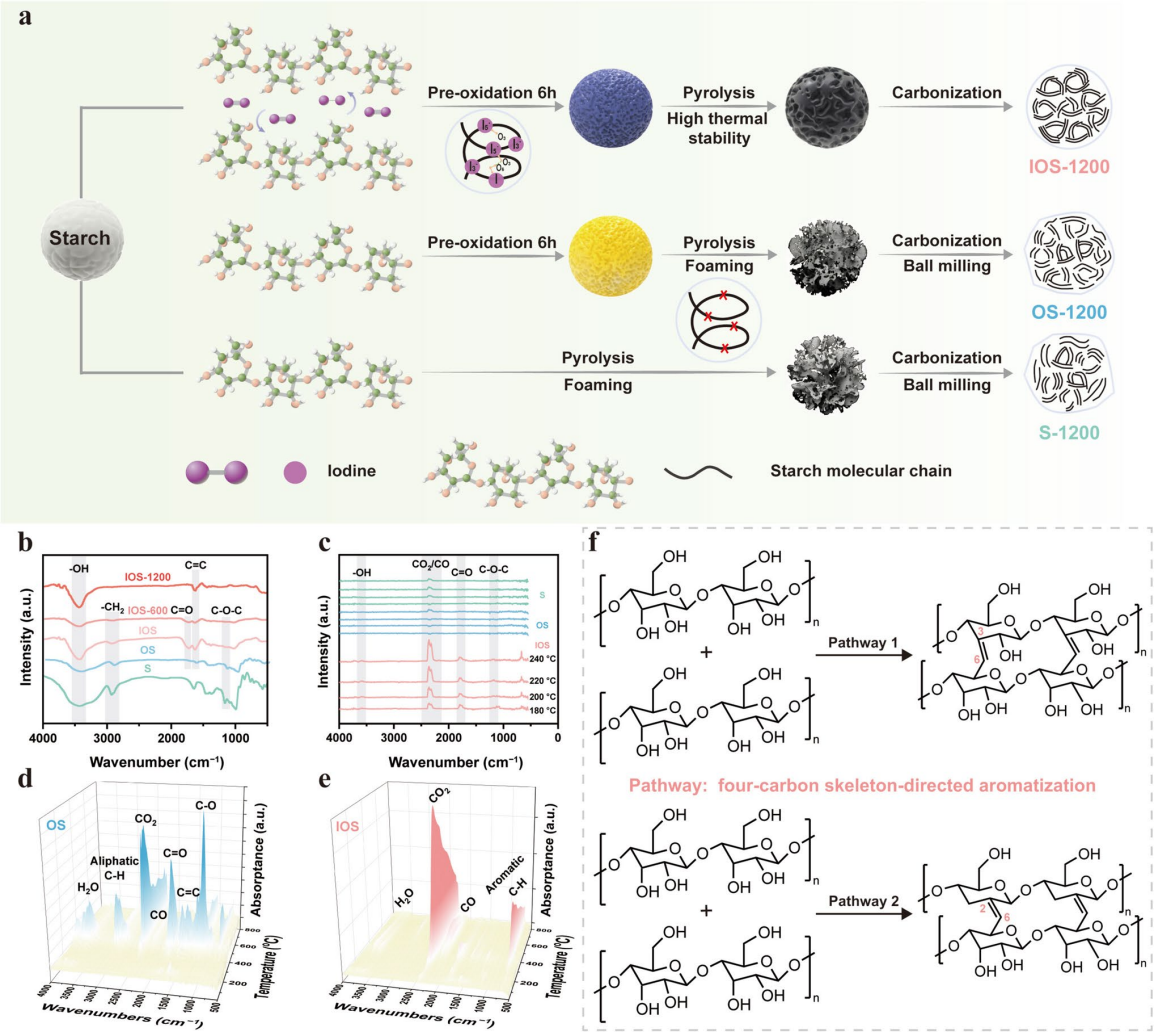
**a** Schematic diagram of the synthesis procedures of S-1200, OS-1200 and IOS-1200. **b** FTIR spectra of S, OS, IOS-600 and IOS-1200. **c** FTIR spectra of gases produced from S, OS, and IOS at varying temperatures. **d–e** TG-FTIR analysis of gas evolution from S, OS, and IOS. **f** Proposed cross-linking pathways of IOS-1200 [22, 33, 44]

Introducing iodine fundamentally alters this stabilization chemistry. Iodine acts as a redox catalyst that promotes oxidative aromatization and interchain cross-linking at the molecular level, thereby converting the S framework into a thermally robust, conjugated network during a short pre-oxidation step (6 h). This iodine-mediated stabilization prevents the formation and volatilization of levoglucosan, enabling the pre-oxidized S (IOS) to retain its structural integrity during carbonization, as evidenced by the stable, non-foaming morphology of IOS-600. Subsequent heating to 1200 °C then drives controlled carbonization into HC (IOS-1200; Fig. S1c, f). The overall synthesis strategy, together with the corresponding control samples, is schematically illustrated in Fig. 1a and the critical stabilization temperature of 205 °C was identified by coupled thermogravimetric analysis–differential scanning calorimetry (TG-DSC) (Fig. S2) [23, 33]. Consistently, iodine-mediated pre-oxidation increases the carbon yield to 45.5%, compared with < 10% for iodine-free samples, directly reflecting its ability to kinetically suppress chain scission and volatilization while promoting carbon-forming reactions (Fig. S3).

The chemical structural evolution of S during oxidation and subsequent thermal treatment was monitored. FTIR shows that S and OS exhibit a characteristic absorption band at 1160 cm^−1^, attributed to C–O–C stretching vibrations of glycosidic linkages, which becomes nearly absent in IOS, indicating suppression of LG formation. Meanwhile, IOS preferentially undergoes oxidation toward carbonyl functionalities, as evidenced by the emergence of a pronounced C=O stretching band at ~ 1730 cm^−1^, which is much less evident in OS. Upon subsequent carbonization, IOS-1200 displays a markedly intensified C=C stretching band at ~ 1630 cm^−1^, confirming enhanced conjugation and aromatization (Fig. 1b). Furthermore, the gaseous products and structural evolution during pyrolysis are characterized using thermogravimetric analysis coupled with TG-FTIR-MS. Figures 1c and S4 show the FTIR spectra of the evolved gases at different temperatures, and Figs. S5 and 1d–e present the corresponding TG-FTIR contour plots. S and OS exhibit distinct bands ~ 2900 to 3000 cm^−1^, assigned to asymmetric C–H stretching, indicating the cleavage of small alkyl fragments or aliphatic side chains during thermal treatment [34, 35]. A strong C=O stretching band at ~ 1750 cm^−1^, characteristic of aldehydes and ketones, reaches maximum intensity at ~ 400 °C and diminishes at higher temperatures due to secondary cracking reactions [36–39]. In contrast, IOS exhibits only very weak signals corresponding to small alkyl fragments across 200–800 °C, indicating that the formation of labile aliphatic species is effectively suppressed in the presence of iodine. Concurrently, relatively enhanced C=O and C=C vibrations (~ 1510 cm^−1^) emerge at 600–800 °C (Figs. 1c and S4f), highlighting the superior thermal robustness of its evolving aromatic framework.

MS-derived evolution profiles were analyzed to resolve the distinct pyrolysis pathways of S, OS, and IOS (Fig. S6). For S, cleavage of inter-unit glycosidic bonds above 280 °C generates LG accompanied by H₂O release (*m/z* 18). The volatilized LG subsequently undergoes ring-opening fragmentation and secondary oxidation to yield aldehydes (*m/z* 29, •CHO^+^), acetyl fragments (*m/z* 43, 43, CH_3_CO^+^), CO (*m/z* 28), and CO_2_ (*m/z* 44), driving severe foaming and poor carbon retention.

In OS, oxygen-containing groups introduced during pre-oxidation partially redirect the pyrolysis chemistry toward a pathway herein referred to as the “hydroxyl–aldehyde–carboxylic acid–anhydride” pathway, as evidenced by intensified MS signals at m/z 29, 43, 44, and 60 (CH_3_COOH^+^) [23, 40]. During this process, aldehydes are further converted into carboxylic acids, while adjacent carboxyl groups undergo dehydration condensation to form anhydride-related intermediates [40]. Although anhydride formation partially suppresses foaming by promoting dehydration and cross-linking reactions, the limited 6 h oxidation duration fails to fully inhibit glycosidic-bond cleavage and LG formation, resulting in competition between the anhydride-forming pathway and LG-driven volatilization, as well as incomplete framework stabilization (Fig. S7).

In contrast, IOS exhibits a fundamentally altered pyrolysis behavior characterized by strongly constrained bond cleavage and suppressed volatilization. Below 300 °C, pronounced attenuation of MS signals at *m/z* 32 (CH_3_OH^+^), 29 (•CHO^+^), and 28 (CO^+^) indicates effective suppression of glycosidic-bond cleavage, LG formation, and its subsequent fragmentation into volatile oxygenated species. Concurrently, only weak signals associated with carboxylic acids (*m/z* 45) and acetyl fragments (*m/z* 43) are detected, demonstrating that the oxygen-functional-group-driven “hydroxyl–aldehyde–carboxylic acid–anhydride” pathway is no longer dominant in IOS. Pronounced I^+^ (*m/z* 127) and I_2_^+^ (*m/z* 254) signals are observed in the temperature range of approximately 230–420 °C, indicating gradual iodate decomposition and the continuous generation of reactive iodine species during early-stage carbonization (Fig. S6l, m) [41, 42]. At the early stage of carbonization (< 400 °C), iodine-mediated pre-oxidation facilitates the stabilization of oxygen within the carbon framework through the formation of embedded carbonyl (C = O) structures, as reflected by the markedly enhanced C = O stretching band in the FTIR spectra (Fig. 1b).

XPS analysis further corroborates the iodine-mediated regulation of the carbon framework (Figs. S8 and S9). The C 1*s* spectra can be deconvoluted into *sp*^2^-hybridized C=C, C–O (ether/hydroxyl), C=O, and O–C=O species, while the O 1*s* spectra consist of contributions from carbonyl oxygen (O-I), hydroxyl/ether oxygen (O-II), and carboxyl/ester oxygen (O-III). Notably, IOS-1200 exhibits the highest proportion of carbonyl functionalities (15.25%, Table S1) among all samples, accompanied by a substantial fraction of retained C–O bonds (13.22%). Such a chemical signature suggests that iodine-mediated pre-oxidation favors the formation of chemically constrained and conjugation-stabilized carbon frameworks enriched with carbonyl functionalities. The pronounced π–π* satellite at 289.89 eV in the C 1*s* spectrum further confirms the formation of an extended conjugated framework (Fig. S9d).

Collectively, the suppressed formation of labile oxygenated fragments revealed by TG–FTIR–MS, together with the C=O-enriched and conjugation-dominated chemical states identified by XPS, indicate a fundamentally altered thermal evolution pathway in IOS. Instead of undergoing the random chain scission and volatilization characteristic of S and OS, the stabilized backbone preferentially undergoes site-specific decarboxylation at the C2 or C3 positions [13, 19, 20]. This process generates C3–C6 (pathway 1) or C2–C6 (pathway 2) four-carbon motifs (Fig. 1f) [22, 23, 44], which serve as key intermediates for the construction of thermally stable conjugated aromatic frameworks. The resulting four-carbon-skeleton-directed aromatization pathway effectively suppresses catastrophic LG-driven volatilization and promotes carbon-framework stabilization during carbonization.

Meanwhile, the evolution of gaseous products plays an important role in regulating the final pore architecture. During carbonization, decomposition of oxygen-containing functional groups releases CO_2_ and CO, whose gradual evolution generates internal nanopores within the constrained carbon framework. In particular, gas-phase FTIR reveals a CO_2_-dominated evolution behavior for IOS, indicating that decarboxylation reactions are the primary devolatilization pathway during carbonization. The continuous release and escape of CO_2_ promote the formation and interconnection of internal nanopores [43], while the iodine-stabilized C=O-rich framework suppresses excessive carbon-layer rearrangement and structural collapse during devolatilization [20]. Consequently, IOS preferentially develops abundant interconnected ultramicropores with confined pore sizes, which later serve as the primary sodium-storage sites in the plateau region.

### Microscopic Structure Analysis

**Fig. 2:**
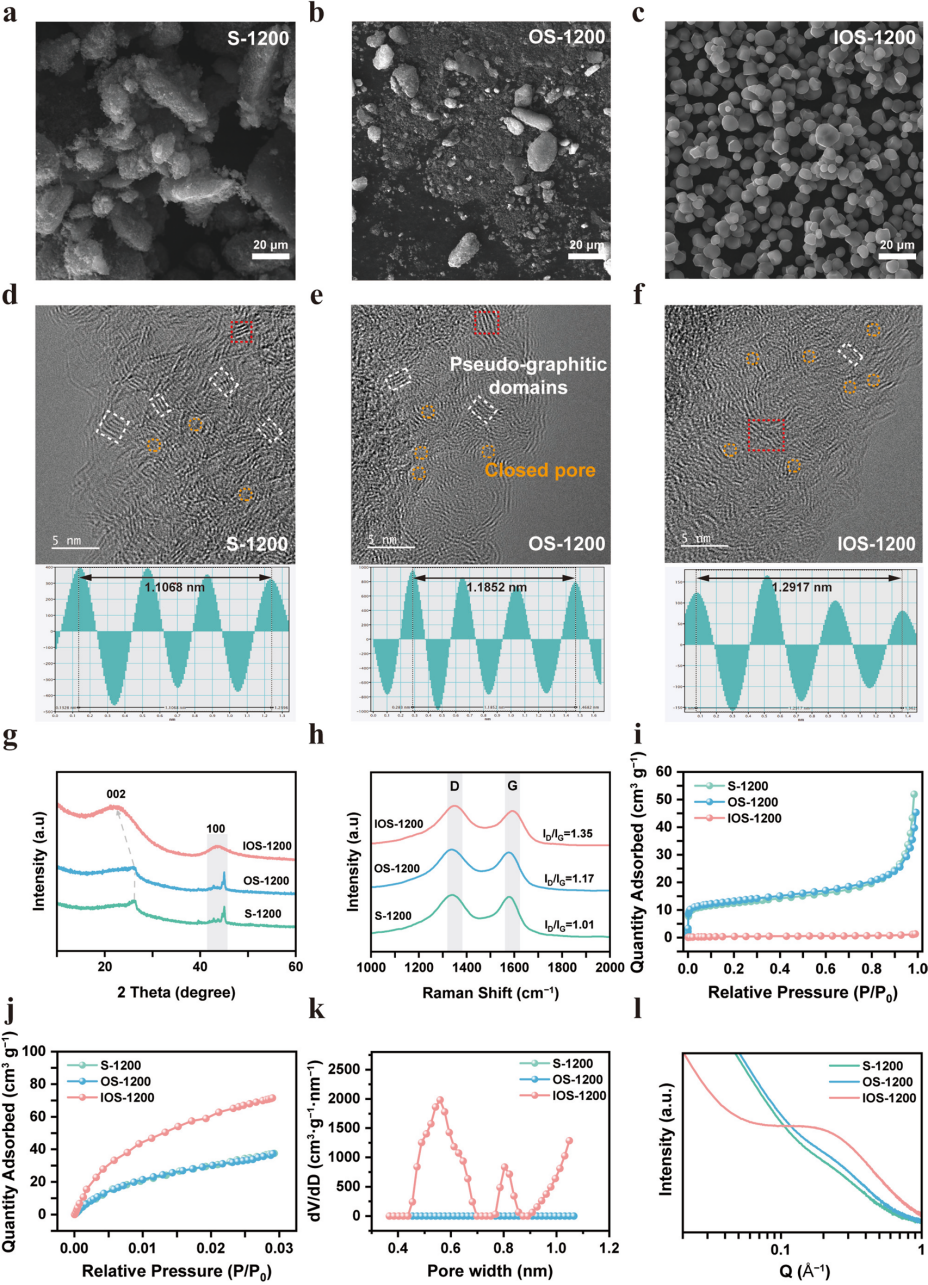
Microscopic structure characterization of S-1200, OS-1200, and IOS-1200. **a–c** SEM images of S-1200, OS-1200 and IOS-1200. **d–f** HRTEM images of S-1200, OS-1200, and IOS-1200 (regions for interlayer spacing measurement are marked by red dashed rectangles). **g** XRD patterns, **h** Raman spectra, **i** N_2_ sorption isotherms, **j** CO_2_ sorption isotherms, and **k** pore-size distributions derived from the CO_2_ adsorption data. **l** SAXS patterns

SEM and HRTEM analyses reveal that, unlike S-1200 and OS-1200, which undergo particle fusion and agglomeration at 1200 °C, IOS-1200 retains its original polyhedral/near-spherical morphology with smooth edges (Figs. 2a–c and S10). At the nanoscale, all samples feature short-range ordered carbon domains within long-range disordered matrices and curved graphitic layers forming pores (Fig. 2d–f). However, IOS-1200 exhibits a higher density of closed nanopores together with locally expanded graphitic layers compared with S-1200 and OS-1200, indicating enhanced structural disorder and enlarged ion-accessible regions, which are favorable for Na^+^ storage and transport.

XRD, Raman spectroscopy and gas adsorption collectively reveal a highly disordered yet functionally optimized carbon framework in IOS-1200. Compared with S-1200 and OS-1200, which show typical turbostratic carbon reflections at ~ 26° and ~ 44°, IOS-1200 exhibits broadened and downshifted (002) and (100) peaks (~ 22.5° and ~ 43.5°) in Fig. 2g, indicative of suppressed crystallinity and pronounced turbostratic disorder. The corresponding interlayer spacing expands to 0.405 nm, markedly larger than those of S-1200 (0.342 nm) and OS-1200 (0.344 nm), which is favorable for lowering the Na^+^ migration barrier [14, 45]. The slight deviation between the XRD-derived d_002_ value and the locally observed HRTEM lattice spacing can be attributed to the distinct measurement principles of the two techniques, where XRD reflects the bulk-averaged interlayer spacing while HRTEM probes localized nanoscale regions with structural heterogeneity. Raman spectra further corroborate this structural evolution (Figs. 2h and S11), with IOS-1200 displaying the highest I_D_/I_G_ ratio (1.35), reflecting increased defect density and structural disorder.

Despite its highly disordered nature, IOS-1200 exhibits an exceptionally low external surface area (1.5 m^2^ g^−1^), as revealed by Type III N_2_ adsorption–desorption isotherms (Fig. 2i), which is expected to suppress electrolyte penetration and mitigate initial irreversible capacity loss. In contrast, CO_2_ adsorption discloses a substantially higher ultramicropore volume (0.144 cm^3^ g^−1^), dominated by pores of 0.4–0.9 nm centered at 0.5–0.6 nm (Fig. 2j, k). Small-angle X-ray scattering (SAXS) measurements further identify a distinct high-q shoulder exclusively in IOS-1200, indicative of abundant closed ultramicropores (Figs. 2l and S12). This unusual combination of expanded disordered carbon layers, ultralow external surface area and abundant closed ultramicropores arises from iodine-mediated regulation of oxygen-functional-group evolution, gas-release behavior, and carbon-framework stabilization during carbonization, thereby providing a structural basis for nanopore-filling-dominated Na^+^ storage with high reversibility in the low-potential plateau region [12, 46].

### Sodium-Storage Performance Analysis

**Fig. 3:**
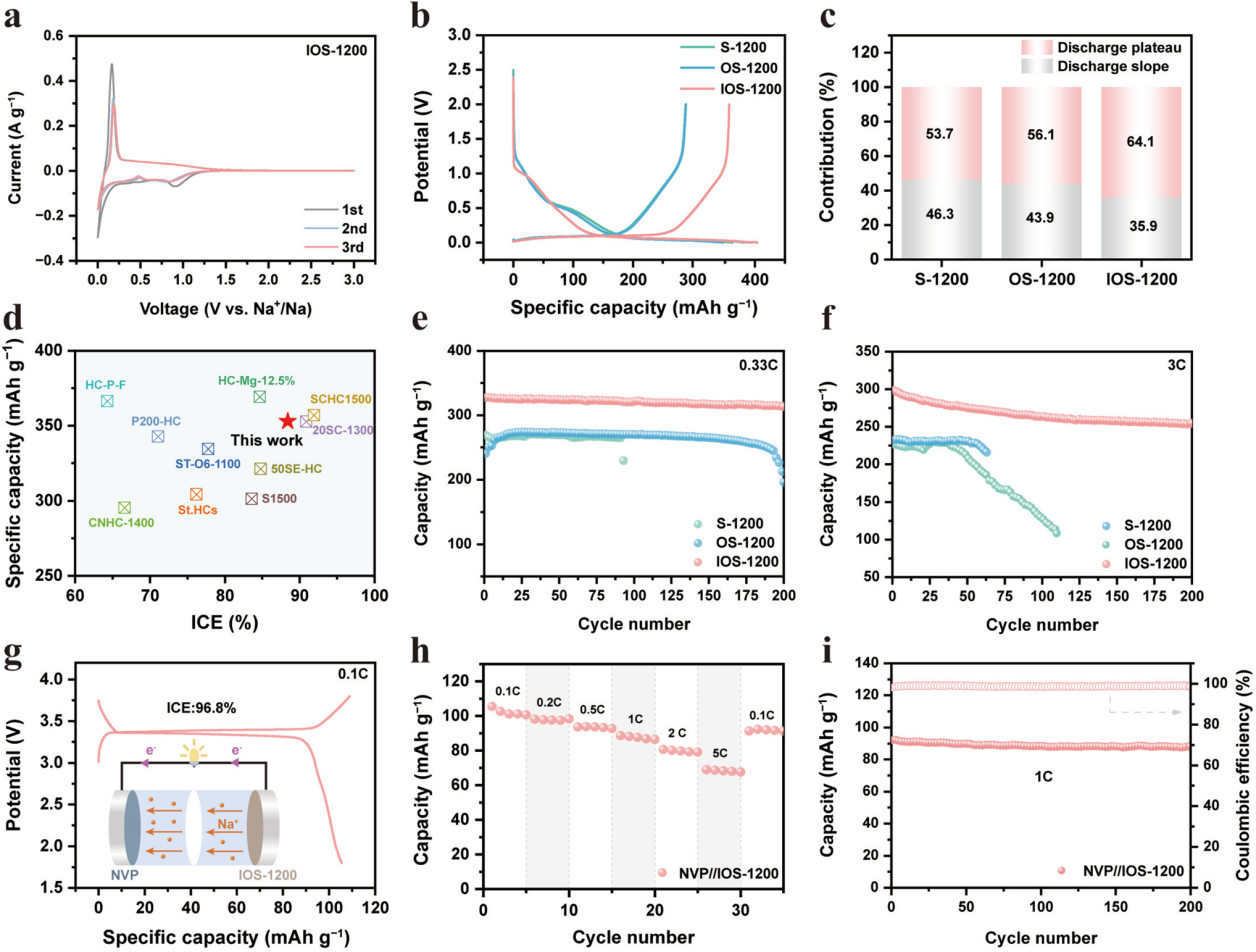
Electrochemical characterization of HC anode materials and the corresponding full cell. **a** CV profiles of IOS-1200 at a scan rate of 0.1 mV s^−1^. **b** GCD curve of HC samples at 0.1C. **c** Contributions of the plateau (< 0.1 V) and slope (> 0.1 V) regions to the discharge specific capacity. **d** Comparative study of the ICE and first-charge specific capacity of IOS-1200 and other reported carbon anodes [23, 40, 47–54]. **e–f** Cycling performance at 0.33C and 3C of HC samples. **g** GCD curve at 0.1C, **h** rate performance and **i** long-cycle test of NVP//IOS-1200 full cell

The electrochemical performance of the HC materials is evaluated in half-cells using sodium metal as the counter electrode. CV reveals markedly suppressed irreversible reactions for IOS-1200, evidenced by a weaker and narrower reduction feature between 0.2 and 1.0 V in the first cycle compared with OS-1200 (Figs. 3a and S13). A pronounced redox peak near 0.1 V is observed for IOS-1200, corresponding to Na^+^ insertion into closed ultramicropores and accounting for its enhanced low-voltage storage contribution. Consistently, GCD profile shows that IOS-1200 delivers a high reversible capacity of 352.9 mAh g^−1^ at 0.1C with an ICE of 88.4%, substantially outperforming OS-1200 (288.6 mAh g^−1^, 74.8%) and S-1200 (287.9 mAh g^−1^, 73.0%) (Fig. 3b and Table S2). Notably, the plateau capacity contributes 64.1% of the discharge capacity for IOS-1200 (Fig. 3c), consistent with efficient Na^+^ filling within abundant ultramicropores. Moreover, IOS-1200 exhibits outstanding rate capability, delivering 288.9 mAh g^−1^ at 5C and fully recovering its initial capacity upon returning to 0.1C (Fig. S14a). This behavior highlights the robust structural stability and fast Na^+^-transport kinetics enabled by the IOS architecture. In comparison with recently reported starch-derived HC anodes, IOS-1200 demonstrates a well-balanced electrochemical performance in terms of initial capacity, ICE and rate capability (Figs. 3d and S14b), highlighting its competitive advantages for practical SIB applications [23, 40, 47–54]. Long-term cycling tests demonstrate the outstanding durability of IOS-1200, which retains 313.3 mAh g^−1^ at 0.33C and 254.5 mAh g^−1^ at 3C after 200 cycles, corresponding to capacity retentions of 95.6% and 85.3%, respectively (Fig. 3e, f). These results highlight the synergistic role of preserved particle morphology, expanded interlayer spacing and strong nanoconfinement in enabling highly reversible and kinetically favorable Na^+^ storage.

To assess practical viability, a full cell was assembled using Na_3_V_2_(PO_4_)_3_ (NVP) as the cathode and IOS-1200 as the anode. The full cell exhibits highly reversible charge–discharge behavior within 1.8–3.8 V (Fig. 3g) and delivers discharge capacities of 105.5, 100.4, 96.1, 91.4, 84.1, and 72.6 mAh g^−1^ at rates from 0.1C to 5C (Fig. 3h). When cycled at 1C, the cell maintains 95.6% of its initial capacity (Fig. 3i), demonstrating excellent rate performance and cycling stability, and underscoring the practical promise of IOS-1200 anode for SIBs.

### Charge-Transfer Kinetics and SEI Layer Evolution

**Fig. 4:**
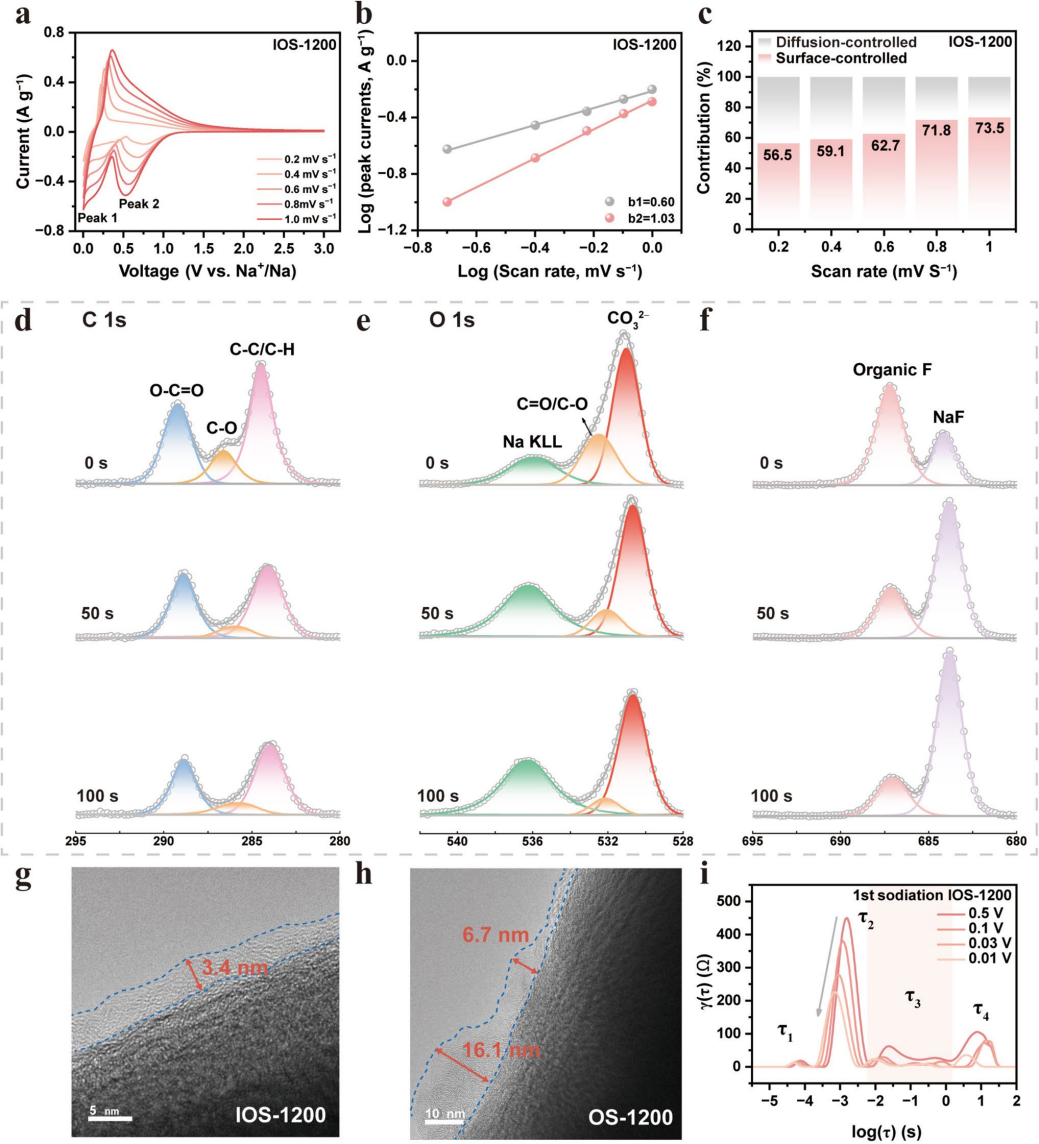
Interfacial kinetic and structural characterization. **a** CV curves of IOS-1200 at different scan rates ranging from 0.2 to 1.0 mV s.^−1^. **b** Corresponding *b*-values determined from linear fitting of the redox peak currents. **c** Capacitive contribution to the total capacity of IOS-1200 at various scan rates. Depth-profiling XPS spectra of the SEI on the IOS-1200 anode after 10 cycles charged to 3.0 V: **d** C 1*s*, **e** O 1*s*, and **f** F 1*s*. HRTEM images of **g** IOS-1200 and **h** OS-1200 anodes after 5 cycles charged to 3.0 V. **i** DRT curves fitted from EIS spectra for IOS-1200

To elucidate the sodium-storage kinetics of the materials, CV analyses were conducted on OS-1200 and IOS-1200. The capacitive contributions were quantified using CV curves collected at scan rates from 0.2 to 1.0 mV s^−1^ (Figs. 4a–c and S15). According to the relationship $i=a{v}^{b}$, the corresponding $b$ values were determined from the slope of the fitted log($i$) versus log($v$) plots [55]. For IOS-1200, the $b$ values of peaks 1 and 2 are 0.60 and 1.03, respectively (Fig. 4b), while OS-1200 shows values of 0.75 and 1.06 (Fig. S15b). These results indicate mixed diffusion–capacitive behavior in the low-voltage plateau region and predominantly capacitive behavior in the high-voltage sloping region. The relative contributions of diffusion-controlled and capacitive processes were further quantified using the equation $i={k}_{1}{v}^{1/2}+{k}_{2}v$, where ${k}_{1}$ and ${k}_{2}$ correspond to diffusion-controlled and surface-controlled contributions, respectively [56]. As shown in Figs. 4c and S15c, OS-1200 consistently exhibits a higher capacitive contribution than IOS-1200 (67.9–82.6% vs. 56.5–73.5%), which can be attributed to its larger specific surface area that facilitates stronger surface adsorption.

To further probe Na^+^ transport, GITT measurements were performed at 0.05C to evaluate the Na^+^ diffusion coefficient (*D*_Na⁺_) during sodiation/desodiation. IOS-1200 exhibits consistently higher *D*_Na⁺_ values than OS-1200 and S-1200 at voltages above 0.1 V (Fig. S16), where Na^+^ storage is dominated by defect adsorption and benefits from the more favorable diffusion kinetics associated with abundant C=O functional groups [18]. However, as the voltage approaches 0.1 V, *D*_Na⁺_ shows a pronounced decrease followed by a rapid increase, corresponding to the sequential Na^+^ intercalation and accumulation processes and the subsequent pore-filling stage, respectively [14].

The chemical composition and depth profile of the SEI are subsequently examined by ex situ XPS after 10 cycles (Figs. 4d–f and S17). In the C 1*s* and O 1*s* regions, signals assigned to C–O, C=O, and O–C=O species originate predominantly from solvent-derived organic components such as ROCO_2_Na, and decay rapidly upon Ar^+^ sputtering, indicating preferential enrichment in the outer SEI. In contrast, the CO_3_^2−^ signal remains pronounced after etching, implying that inorganic carbonates (e.g., Na_2_CO_3_) constitute the SEI framework. Notably, IOS-1200 shows a much stronger Na–F signal in the F 1*s* region after sputtering, consistent with extensive PF_6_^−^ anion decomposition and the formation of a NaF-rich inner layer (Fig. 4f). OS-1200 and S-1200, however, display weak Na–F signals even after sputtering, indicating limited PF_6_^−^ participation and a predominantly organic SEI (Fig. S17e, f). High-resolution TEM characterization of the cycled electrodes after 5 cycles further corroborates these results, revealing a thinner and more uniform SEI on IOS-1200, whereas OS-1200 develops a thicker and less homogeneous film (Fig. 4g, h). The reduced thickness and enhanced continuity on IOS-1200 are consistent with suppressed solvent decomposition and the formation of a mechanically robust inorganic inner layer.

To assess the kinetic consequences of this SEI chemistry, in situ EIS coupled with distribution of relaxation times (DRT) analysis was performed. During the initial sodiation and desodiation process, IOS-1200 exhibits significantly lower impedance than OS-1200 (Fig. S18), reflecting greatly accelerated interfacial kinetics. DRT analysis reveals four characteristic processes: τ_1_ (~ 10^−5^–10^−4^ s) for contact resistance (R_s_), τ_2_ (~ 10^−4^–10^−2^ s) for ion migration through the SEI (R_SEI_), τ_3_ (~ 10^−2^–1 s) for charge-transfer resistance (R_ct_), and τ_4_ (> 1 s) for solid-state Na⁺ diffusion [57, 58]. IOS-1200 maintains substantially lower R_SEI_ and R_ct_ throughout sodiation from 0.50 to 0.01 V (Figs. 4i and S19), reflecting facilitated ion transport through the inorganic-rich SEI. In contrast, OS-1200 shows only modest changes in R_SEI_ and nearly constant R_ct_, consistent with a discontinuous, organic-rich SEI that offers limited kinetic benefit.

**Fig. 5:**
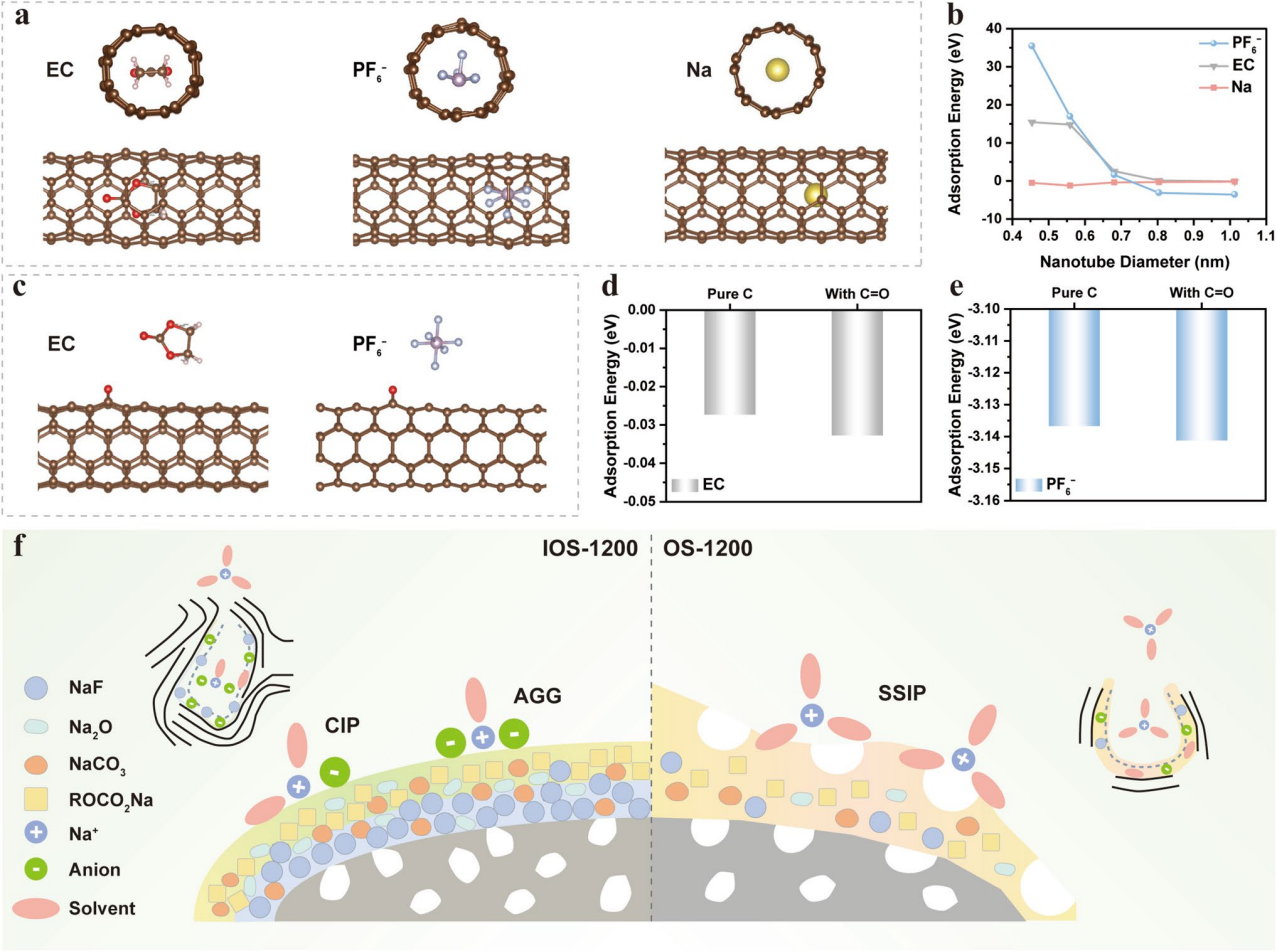
Theoretical Calculations and Schematic Illustrations of SEI. **a** Schematic adsorption model of EC, and PF_6_^−^ and Na on a CNT. **b** Corresponding calculated adsorption energies across CNTs with different diameters. **c** Schematic adsorption model of EC and PF_6_^−^ on a C=O-functionalized carbon surface, along with the corresponding adsorption energies on **d** the pure and **e** functionalized surfaces. **f** Schematic illustration of the distinct SEI structures. The mechanism guided by ultramicropores and C=O functionalities in IOS-1200 (left) produces a uniform SEI with an inorganic-rich inner layer, while OS-1200 (right) forms an uneven, predominantly organic SEI

The pronounced differences in SEI composition can be attributed to the unique ultramicroporous, C=O-rich interfaces generated by iodine-catalyzed cross-linking in IOS-1200, whose porosity is dominated by pores of 0.4–0.9 nm centered at 0.5–0.6 nm. To elucidate how such ultramicropore confinement regulates interfacial solvation and SEI formation, DFT calculations were carried out using CNT models with diameters of 0.484, 0.559, 0.681, 0.804, and 1.013 nm to simulate pores of different sizes. The adsorption energies of representative electrolyte components (Na^+^, ethylene carbonate (EC) and PF_6_^−^) were systematically evaluated (Fig. 5a, b). The calculations show that EC molecules are sterically excluded and cannot be spontaneously adsorbed when the pore diameter is below 0.804 nm, whereas Na^+^ preserves strong intrinsic adsorption across all examined pore sizes. This indicates that sub-0.804-nm pores effectively block solvated species while remaining accessible to the small-radius Na^+^, so that ultramicropores act as molecular sieves that promote the transition of interfacial solvation from solvent-rich configurations to anion-enriched contact ion pairs (CIPs) and aggregates (AGGs) [16, 59]. In parallel, PF_6_^−^ adsorption becomes energetically more favorable than EC once the pore diameter exceeds 0.681 nm, further enhancing anion enrichment within sufficiently wide ultramicropores.

To assess the contribution of surface chemistry, additional DFT calculations were performed on C=O-functionalized CNT models (Figs. 5c–e and S20). The C=O-decorated surfaces exhibit significantly stronger adsorption toward PF_6_^−^ than toward EC, indicating that oxygen-containing functionalities further promote anion-enriched interfacial environments and favor anion-derived interfacial reactions.

Taken together, ultramicropore-induced size-selective ion sieving, coupled with strong PF_6_^−^ binding at C=O-rich interfaces, is expected to create an anion-enriched interfacial microenvironment that favors PF_6_^−^-derived inorganic interphases on the external surface and potentially within nanoconfined pore regions (Fig. 5f). Such inorganic-rich interphases can substantially lower the interfacial energy barrier, thereby enabling fast Na^+^ transport, high ICE and superior rate capability.

### Sodium-Storage Mechanism Analysis

**Fig. 6:**
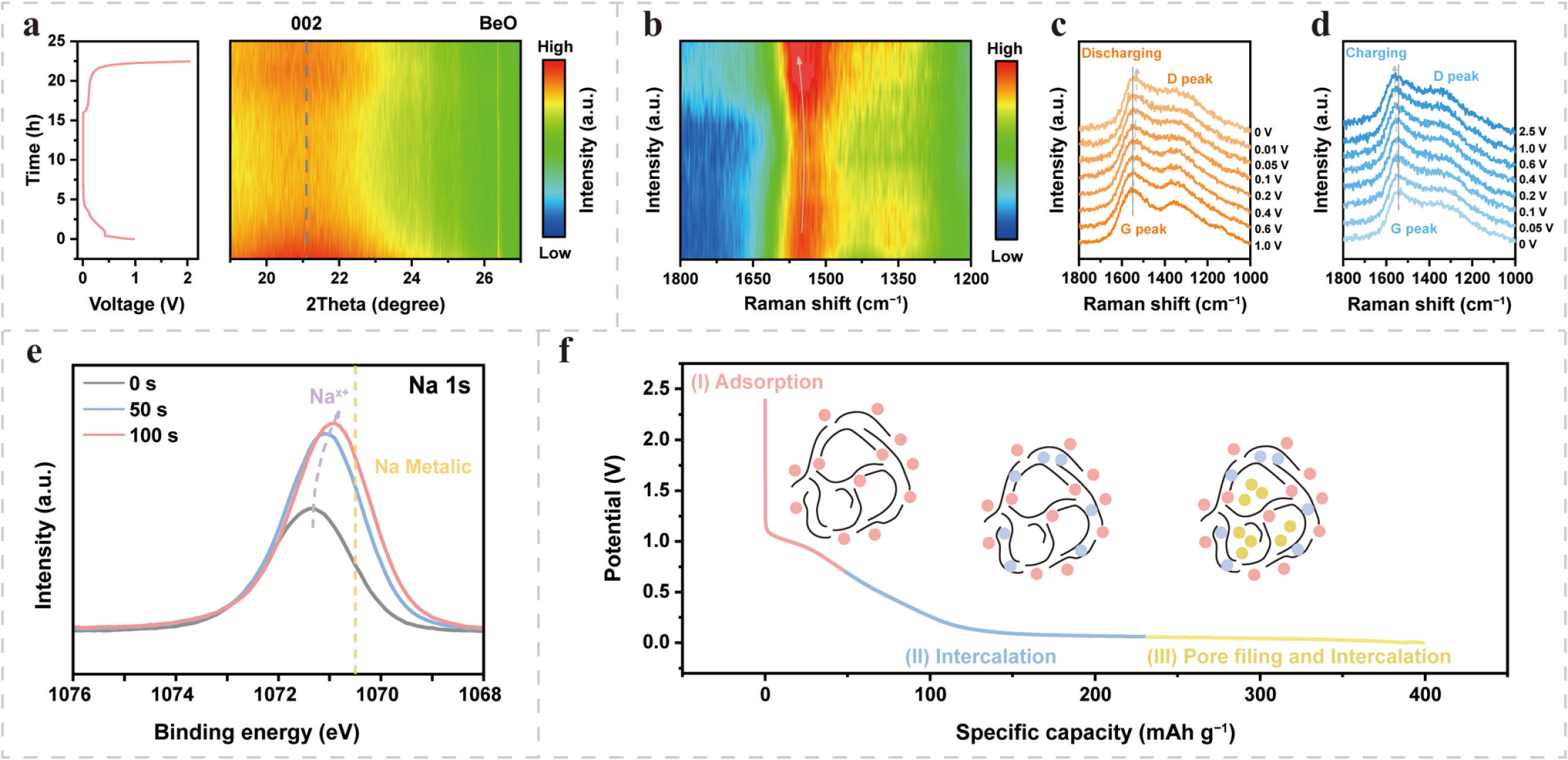
Sodium-storage mechanism of IOS-1200. **a** In situ XRD patterns during the initial cycle. **b** In situ Raman spectra collected during the initial cycle, and selective in situ Raman spectra during **c** discharge process and **d** charge process. **e** Ex situ XPS spectra of the Na 1*s* region in the fully sodiated (0 V) states. **f** Schematic illustration of the sodium-ion storage mechanism

In situ XRD and Raman spectroscopy were employed to elucidate the structural evolution of IOS-1200 during sodium storage. As shown in Figs. 6a and S21, the nearly unchanged (002) peak during the first cycle indicates robust structural stability, which accounts for the excellent long-term cycling performance of IOS-1200. The in situ Raman spectra (Fig. 6b–d) exhibit the characteristic D and G bands. During the discharge process, the attenuation of the D-band intensity reveals that Na^+^ is preferentially adsorbed at surface and defect sites [60, 61]. As the potential decreases to 0.6 V, the decrease in intensity and broadening of the D band without a noticeable shift suggest that Na^+^ storage in the sloping-voltage region is dominated by adsorption onto C=O active sites and structural defects [62]. Meanwhile, an evident red shift in the G band emerges at 0.6 V, indicative of Na^+^ insertion into the carbon layers and occupation of π* antibonding orbitals [59, 63]. In the plateau region from 0.1 to 0 V, the G band further shifts toward lower frequencies due to enhanced electron occupation in the π* band of the carbon framework, resulting in strong electron–phonon coupling and the formation of Na–C interactions [13, 14]. Concurrently, the near disappearance of the D band suggests effective passivation of carbon-surface defects through the generation of Na clusters. During the subsequent charging process, both the D-band intensity and G-band position recover to their initial states, confirming the highly reversible Na⁺ storage mechanism in IOS-1200.

Ex situ XPS was conducted on IOS-1200 electrodes in the fully sodiated states to clarify the chemical states of stored sodium (Fig. 6e). The Na 1*s* peak at ~ 1071 eV, originating from SEI-derived Na–O–C species [64, 65], shifts slightly to lower binding energy with sputtering while metallic Na remains undetected, evidencing the coexistence of Na^+^ and quasi-metallic sodium. To further support this conclusion, fully sodiated electrodes at different potentials were immersed in a 1% phenolphthalein–alcohol solution. As shown in Fig. S22, electrodes discharged below 0.01 V exhibit a distinct red coloration, evidencing Na cluster formation during the pore-filling process under the low-potential plateau. Upon charging, the color fades, demonstrating the high reversibility of Na cluster formation. Consequently, the sodium-storage mechanism of the IOS-1200 anode can be classified into three stages (Fig. 6f). Stage I (2.5–0.6 V): Na^+^ adsorption dominates, involving external surface sites (open pores and edge defects) and internal surface sites associated with structural defects and C=O functional groups. Stage II (0.6–0.01 V): Na^+^ intercalation into graphitic layers occurs continuously, accompanied by the progressive formation of NaC_x_ species. Stage III (0.01–0 V): the abundant ultramicropores induce a pore-filling process at the low-potential plateau, leading to the formation of quasi-metallic sodium clusters.

## Conclusions

In summary, starch-derived spherical HC (IOS-1200) was synthesized via an iodine-mediated oxidative cross-linking strategy, producing abundant ultramicropores (< 0.9 nm) and C=O functionalities. The ultramicropores function as nanoconfining sieves, regulating the electrolyte solvation structure into anion-enriched CIPs/AGGs and favoring the formation of inorganic-rich interphases within nanoconfined pore regions. Meanwhile, ultramicropores wider than 0.681 nm, together with C=O groups, promote favorable PF_6_^−^ adsorption on pore surfaces, further contributing to anion-derived interfacial chemistry. Additionally, C=O groups act as highly reversible Na^+^ adsorption sites, enabling fast charge-transfer kinetics in the sloping-voltage region. Benefiting from these synergistic desolvation, nanoconfinement, and interfacial-regulation effects, IOS-1200 achieves excellent sodium-storage performance in ester-based electrolytes, including a high ICE (88.4%), high reversible capacity (352.9 mAh g^−1^ at 0.1C), outstanding rate capability (288.9 mAh g^−1^ at 5C), and stable cycling performance (95.6% retention at 0.33C after 200 cycles). These findings provide a promising materials-design strategy for high-performance SIB anodes.

## Supplementary Information

Below is the link to the electronic supplementary material.

Supplementary file1 (DOCX 7467 KB)

## References

[1] D. Saurel, B. Orayech, B. Xiao, D. Carriazo, X. Li et al., From charge storage mechanism to performance: a roadmap toward high specific energy sodium-ion batteries through carbon anode optimization. Adv. Energy Mater. **8**(17), 1703268 (2018). 10.1002/aenm.201703268

[2] X. Chen, C. Liu, Y. Fang, X. Ai, F. Zhong et al., Understanding of the sodium storage mechanism in hard carbon anodes. Carbon Energy **4**(6), 1133–1150 (2022). 10.1002/cey2.196

[3] J. Zheng, W. Liu, S. Li, Y. Lai, J. Li et al., Spatial confinement strategy modulated by kinetic diameters of gaseous molecules for sodium storage. Energy Storage Mater. **73**, 103835 (2024). 10.1016/j.ensm.2024.103835

[4] J.H. Li, Y.B. Zhang, Y.R. Jia, C.X. Yang, Y. Chu et al., Progress and challenges in the use of carbon anodes for high-energy and fast-charging sodium-ion batteries. New Carbon Mater. **39**(5), 729–742 (2024). 10.1016/S1872-5805(24)60870-X

[5] D. Ma, Z. Zhao, Y. Wang, X. Yang, M. Yang et al., Unlocking the design paradigm of in-plane heterojunction with built-in bifunctional anion vacancy for unexpectedly fast sodium storage. Adv. Mater. **36**(4), 2310336 (2024). 10.1002/adma.202310336 10.1002/adma.202310336 38009638

[6] K.Y. Liang, Y.B. Guan, B. Qiu, L.D. Wang, H.D. Wei et al., Expansion-induced spontaneous polarization facilitates sodium-ion storage in red phosphorus anode. Rare Met. **44**(12), 9975–9985 (2025). 10.1007/s12598-025-03588-1

[7] S. Luo, Z. Hou, Z. Qi, Q. Li, Y. Pang et al., Insights into targeted effects of salt and solvent on Na^+^ storage in hard carbon anodes toward ultra-low temperature sodium batteries. Sci. China Chem. **69**(3), 1425–1433 (2026). 10.1007/s11426-025-2947-2

[8] J.-T. Li, N. Sawut, Y.-C. Zhao, P. Liu, Y.-X. Wang et al., Microstructure-mechanism-performance relationships in hard carbon anode materials for sodium-ion batteries. New Carbon Mater. **40**(4), 860–868 (2025). 10.1016/S1872-5805(25)61023-7

[9] Z. Chen, J. Shen, W. Deng, Y. Huang, P. Shan et al., Achieving over 200 Wh kg^–1^ sodium-ion pouch cell by quantitative engineering of hard carbon pores. Natl. Sci. Rev. **13**(3), nwaf566 (2026). 10.1093/nsr/nwaf566 41660283 10.1093/nsr/nwaf566 PMC12875110

[10] W. Deng, Y. Wang, Z. Chen, H. Huang, C. Chen et al., Catalyst-assisted chemical vapor deposition engineering of hard carbon with abundant closed pores for high-performance sodium-ion batteries. Adv. Funct. Mater. **35**(41), 2501721 (2025). 10.1002/adfm.202501721

[11] J.M. Stratford, A.K. Kleppe, D.S. Keeble, P.A. Chater, S.S. Meysami et al., Correlating local structure and sodium storage in hard carbon anodes: insights from pair distribution function analysis and solid-state NMR. J. Am. Chem. Soc. **143**(35), 14274–14286 (2021). 10.1021/jacs.1c06058 34431677 10.1021/jacs.1c06058

[12] Y. Li, A. Vasileiadis, Q. Zhou, Y. Lu, Q. Meng et al., Origin of fast charging in hard carbon anodes. Nat. Energy **9**(2), 134–142 (2024). 10.1038/s41560-023-01414-5

[13] Z. Song, M. Di, X. Zhang, Z. Wang, S. Chen et al., Nanoconfined strategy optimizing hard carbon for robust sodium storage. Adv. Energy Mater. **14**(43), 2401763 (2024). 10.1002/aenm.202401763

[14] J. Duan, Z. Xu, M. Li, P. Yang, H. Hu et al., Structure regulation of hard carbon with enriched semi-closed ultramicropores for enhanced rapid sodium storage. Adv. Funct. Mater. **35**(46), 2508822 (2025). 10.1002/adfm.202508822

[15] J. Zheng, C. Guan, H. Li, D. Wang, Y. Lai et al., Unveiling the microscopic origin of irreversible capacity loss of hard carbon for sodium-ion batteries. Adv. Energy Mater. **14**(15), 2303584 (2024). 10.1002/aenm.202303584

[16] Y. Zhang, S.-W. Zhang, Y. Chu, J. Zhang, H. Xue et al., Redefining closed pores in carbons by solvation structures for enhanced sodium storage. Nat. Commun. **16**, 3634 (2025). 10.1038/s41467-025-59022-8 40240373 10.1038/s41467-025-59022-8 PMC12003850

[17] M. Liu, F. Wu, Y. Gong, Y. Li, Y. Li et al., Interfacial-catalysis-enabled layered and inorganic-rich SEI on hard carbon anodes in ester electrolytes for sodium-ion batteries. Adv. Mater. **35**(29), 2370207 (2023). 10.1002/adma.202370207 10.1002/adma.202300002 37018163

[18] Y. Sun, D. Zuo, C. Xu, B. Peng, J.C. Li et al., A “grafting technique” to tailor the interfacial behavior of hard carbon anodes for stable sodium-ion batteries. Energy Environ. Sci. **18**(4), 1911–1919 (2025). 10.1039/D4EE05305B

[19] P. Wang, B. Wang, Y. Li, W. Wang, Y. Sun et al., Selecting the molecular components of a pitch to produce a hard carbon anode with a high sodium storage capacity. New Carbon Mater. **41**(1), 142–155 (2026). 10.1016/S1872-5805(25)60971-1

[20] Y. Chai, J. Guo, C. Hong, Z. Yi, W. Li et al., Reducing steric hindrance to enhance the oxidation reactivity of coal precursors for high-performance hard carbons in sodium-ion batteries. Energy Storage Mater. **83**, 104678 (2025). 10.1016/j.ensm.2025.104678

[21] Y. Wang, W. Li, C. Huang, Z. Jiang, F. Liu et al., High-crystallinity wrinkled hard carbon *via* molecular-level anchoring glucose molecules with cooperative-assembly of intermolecular hydrogen bonds for sodium-ion batteries. Adv. Funct. Mater. **35**(17), 2570102 (2025). 10.1002/adfm.202570102

[22] M.M. Tang, R. Bacon, Carbonization of cellulose fibers: I. Low temperature pyrolysis. Carbon **2**(3), 211–220 (1964). 10.1016/0008-6223(64)90035-1

[23] J. Huang, E. Li, B. Dai, T. Lu, J. Teng et al., Regulating the active hydroxyl group of starch: revealing the evolution of hard carbon structure and sodium storage behavior. Carbon **229**, 119527 (2024). 10.1016/j.carbon.2024.119527

[24] Z. Wen, R. Zhao, T. Tian, T. Zhang, X. Wang et al., Molecular stitching in polysaccharide precursor for fabricating hard carbon with ultra-high plateau capacity of sodium storage. Adv. Mater. **37**(18), 2420251 (2025). 10.1002/adma.202420251 10.1002/adma.202420251 40125840

[25] M.X. Song, L.J. Xie, J.Y. Cheng, Z.L. Yi, G. Song et al., Insights into the thermochemical evolution of maleic anhydride-initiated esterified starch to construct hard carbon microspheres for lithium-ion batteries. J. Energy Chem. **66**, 448–458 (2022). 10.1016/j.jechem.2021.08.050

[26] G. Kresse, J. Furthmüller, Efficiency of ab-initio total energy calculations for metals and semiconductors using a plane-wave basis set. Comput. Mater. Sci. **6**(1), 15–50 (1996). 10.1016/0927-0256(96)00008-0 10.1103/physrevb.54.11169 9984901

[27] J.P. Perdew, K. Burke, M. Ernzerhof, Generalized gradient approximation made simple. Phys. Rev. Lett. **77**(18), 3865–3868 (1996). 10.1103/physrevlett.77.3865 10062328 10.1103/PhysRevLett.77.3865

[28] P. Sun, Z. Qiao, X. Dong, R. Jiang, Z.-T. Hu et al., Designing 3d transition metal cation-doped MRuO_*x*_ as durable acidic oxygen evolution electrocatalysts for PEM water electrolyzers. J. Am. Chem. Soc. **146**(22), 15515–15524 (2024). 10.1021/jacs.4c04096 38785086 10.1021/jacs.4c04096

[29] H.J. Monkhorst, J.D. Pack, Special points for Brillouin-zone integrations. Phys. Rev. B **13**(12), 5188–5192 (1976). 10.1103/physrevb.13.5188

[30] Z. Qiao, R. Jiang, H. Xu, D. Cao, X.C. Zeng, A general descriptor for single-atom catalysts with axial ligands. Angew. Chem. Int. Ed. **63**(40), e202407812 (2024). 10.1002/anie.202407812 10.1002/anie.202407812 38771728

[31] S. Grimme, J. Antony, S. Ehrlich, H. Krieg, A consistent and accurate *ab initio* parametrization of density functional dispersion correction (DFT-D) for the 94 elements H-Pu. J. Chem. Phys. **132**(15), 154104 (2010). 10.1063/1.3382344 20423165 10.1063/1.3382344

[32] Z. Hou, Y. Zhao, Y. Du, F. Wu, W. He et al., Expediting sodium energy of hard carbon by cation/anion co-interfering chemistry. Adv. Funct. Mater. **35**(36), 2505468 (2025). 10.1002/adfm.202505468

[33] Y. Shen, Y. Wang, H. Li, Y.S. He, Z.F. Ma et al., Air-stabilization creates non-crosslinked starch particles for high-performance hard carbon anodes. Adv. Funct. Mater. **36**(1), e09126 (2026). 10.1002/adfm.202509126

[34] S. Yao, K. Zhang, K. Jiao, W. Hu, Evolution of coal structures: FTIR analyses of experimental simulations and naturally matured coals in the Ordos Basin, China. Energy Explor. Exploit. **29**(1), 1–19 (2011). 10.1260/0144-5987.29.1.1

[35] X.K. Shan, S.L. Zhao, Y.Y. Ma, W. Mo, X.Y. Wei, Analysis of pyrolysis performance and molecular structure of five kinds of low-rank coals in Xinjiang based on the TG-DTG method. ACS Omega **7**(10), 8547–8557 (2022). 10.1021/acsomega.1c06350 35309428 10.1021/acsomega.1c06350 PMC8928551

[36] P. Saires, C.A. Barraza, M. Bertero, R. Pujro, M. Falco et al., Characterization of pyrolytic tars derived from different biomasses. Processes **12**(4), 817 (2024). 10.3390/pr12040817

[37] L. Zhang, S. Qi, N. Takeda, S. Kudo, J. Hayashi et al., Characteristics of gas evolution profiles during coal pyrolysis and its relation with the variation of functional groups. Int. J. Coal Sci. Technol. **5**(4), 452–463 (2018). 10.1007/s40789-017-0175-0

[38] F. Zhao, Z. Yang, L. Zhang, C. Zhang, T. Wang et al., The effect of temperature on pyrolysis products during oil shale thermal decomposition. Sci. Rep. **15**, 26135 (2025). 10.1038/s41598-025-11050-6 40681670 10.1038/s41598-025-11050-6 PMC12274543

[39] J. Zheng, Y. Wu, C. Guan, D. Wang, Y. Lai et al., Lignin-derived hard carbon anode with a robust solid electrolyte interphase for boosted sodium storage performance. Carbon Energy **6**(9), e538 (2024). 10.1002/cey2.538

[40] J. Huang, S. Liu, E. Li, B. Dai, T. Lu et al., Double functionalization strategy: using acetate metal salt as medium to optimize hard carbon. Carbon **234**, 119981 (2025). 10.1016/j.carbon.2024.119981

[41] E.A. Pillar-Little, M.I. Guzman, J.M. Rodriguez, Conversion of iodide to hypoiodous acid and iodine in aqueous microdroplets exposed to ozone. Environ. Sci. Technol. **47**(19), 10971–10979 (2013). 10.1021/es401700h 23987087 10.1021/es401700h

[42] K. Kim, B. Kim, Y.-Y. Ahn, K.D. Tran, H.T.M. Truong et al., Production of molecular iodine *via* a redox reaction between iodate and organic compounds in ice. J. Phys. Chem. A **127**(12), 2830–2838 (2023). 10.1021/acs.jpca.3c00482 36919929 10.1021/acs.jpca.3c00482

[43] J. Chen, X. Chen, B. Xu, L. Chen, C. Wang et al., Atomistic mechanisms of oxygen-containing functional groups in hard carbon precursors for enhanced sodium storage performance. Energy Storage Mater. **85**, 104886 (2026). 10.1016/j.ensm.2026.104886

[44] H. Yamamoto, S. Muratsubaki, K. Kubota, M. Fukunishi, H. Watanabe et al., Synthesizing higher-capacity hard-carbons from cellulose for Na- and K-ion batteries. J. Mater. Chem. A **6**(35), 16844–16848 (2018). 10.1039/c8ta05203d

[45] Z. Hong, Y. Zhen, Y. Ruan, M. Kang, K. Zhou et al., Rational design and general synthesis of S-doped hard carbon with tunable doping sites toward excellent Na-ion storage performance. Adv. Mater. **30**(29), 1802035 (2018). 10.1002/adma.201802035 10.1002/adma.201802035 29808566

[46] Y. Youn, B. Gao, A. Kamiyama, K. Kubota, S. Komaba et al., Nanometer-size Na cluster formation in micropore of hard carbon as origin of higher-capacity Na-ion battery. Npj Comput. Mater. **7**, 48 (2021). 10.1038/s41524-021-00515-7

[47] H. Zhong, Q. Huang, M. Zou, F. Li, Y. Liu et al., From food to hard carbon: citric acid enhanced biomass-derived anodes for high-performance sodium storage. Chem. Eng. J. **508**, 160879 (2025). 10.1016/j.cej.2025.160879

[48] Y. Sun, T. Shen, Z. He, S. Wang, Crosslinking modification of starch improves the structural stability of hard carbon anodes for high-capacity sodium storage. J. Colloid Interface Sci. **678**, 1142–1150 (2025). 10.1016/j.jcis.2024.09.191 39341145 10.1016/j.jcis.2024.09.191

[49] G. Yang, J. Zhang, Z. Zhang, X. Qin, Q. Teng et al., Surface functionalized porous spherical hard carbon material derived from taro starch for high performance sodium storage. Electrochim. Acta **521**, 145935 (2025). 10.1016/j.electacta.2025.145935

[50] S. Xu, W. Liu, S. Mao, Y. Wu, B. Yuan et al., *Lotus* root starch derived sustainable hard carbon for fast-charging sodium-ion batteries. Chem. Eng. J. **519**, 165014 (2025). 10.1016/j.cej.2025.165014

[51] J. Zhao, X. Liu, H. Yuan, S. Feng, K. Xiong et al., Insights into the microstructure evolution of starch-derived hard carbon for sodium-ion battery. J. Power. Sources **647**, 237367 (2025). 10.1016/j.jpowsour.2025.237367

[52] J. Xie, H. Ai, K. Du, R. Lv, Regulating ultramicropores and closed-pores in starch-derived hard carbons for efficient sodium ion storage. Carbon **247**, 121077 (2026). 10.1016/j.carbon.2025.121077

[53] L. Chen, L. Zhang, Y. Jiang, J. Zhao, F. Xu et al., Regulation of surface oxygen functional groups in starch-derived hard carbon *via* pre-oxidation: a strategy for enhanced sodium storage performance. Mater. Today Chem. **41**, 102314 (2024). 10.1016/j.mtchem.2024.102314

[54] H. Zeng, J. Zhang, J. He, Q. Zeng, N. Li et al., Starch-derived N-doped hard carbons for sodium-ion storage: preparation and enhanced electrochemical performance. J. Power. Sources **654**, 237850 (2025). 10.1016/j.jpowsour.2025.237850

[55] W. Chen, M. Wan, Q. Liu, X. Xiong, F. Yu et al., Heteroatom-doped carbon materials: synthesis, mechanism, and application for sodium-ion batteries. Small Meth. **3**(4), 1800323 (2019). 10.1002/smtd.201800323

[56] V. Augustyn, P. Simon, B. Dunn, Pseudocapacitive oxide materials for high-rate electrochemical energy storage. Energy Environ. Sci. **7**(5), 1597 (2014). 10.1039/c3ee44164d

[57] P. Wang, S. Xu, S. Wang et al., Unlocking interlayer confinement enables all-slope hard carbon with ultrafast and highly reversible sodium storage. ACS Nano **19**(44), 38735–38748 (2025). 10.1021/acsnano.5c14641 41159759 10.1021/acsnano.5c14641

[58] Y. Zhu, Z. Lao, M. Zhang, T. Hou, X. Xiao et al., A locally solvent-tethered polymer electrolyte for long-life lithium metal batteries. Nat. Commun. **15**, 3914 (2024). 10.1038/s41467-024-48078-7 38724546 10.1038/s41467-024-48078-7 PMC11082227

[59] Y. He, F. Yu, K. Liu, L. Bai, Y. Liu et al., Tuning hard carbon pores at the ångstrom scale facilitates sodium-ion pre-desolvation in high-performance sodium-ion batteries. Adv. Energy Mater. **16**(5), e04760 (2026). 10.1002/aenm.202504760

[60] X. Feng, F. Wu, Y. Li, Y. Fu, Y. Li et al., Critical role of ultra-microporous tunnel structure within hard carbon in boosting sodium-ion storage. Adv. Mater. **38**(1), e01779 (2026). 10.1002/adma.202501779 40937944 10.1002/adma.202501779

[61] Q. Li, X. Liu, Y. Tao, J. Huang, J. Zhang et al., Sieving carbons promise practical anodes with extensible low-potential plateaus for sodium batteries. Natl. Sci. Rev. **9**(8), 084 (2022). 10.1093/nsr/nwac084 10.1093/nsr/nwac084 PMC9385462 35992230

[62] Z. Song, M. Di, S. Chen, Y. Bai, Three-dimensional N/O co-doped hard carbon anode enabled superior stabilities for sodium-ion batteries. Chem. Eng. J. **470**, 144237 (2023). 10.1016/j.cej.2023.144237

[63] J. Yan, Y. Zhang, P. Kim, A. Pinczuk, Electric field effect tuning of electron-phonon coupling in graphene. Phys. Rev. Lett. **98**(16), 166802 (2007). 10.1103/physrevlett.98.166802 17501446 10.1103/PhysRevLett.98.166802

[64] Y. Zeng, J. Yang, H. Yang, Y. Yang, J. Zhao, Bridging microstructure and sodium-ion storage mechanism in hard carbon for sodium ion batteries. ACS Energy Lett. **9**(3), 1184–1191 (2024). 10.1021/acsenergylett.3c02751

[65] Z. Wang, X. Feng, Y. Bai, H. Yang, R. Dong et al., Probing the energy storage mechanism of quasi-metallic Na in hard carbon for sodium-ion batteries. Adv. Energy Mater. **11**(11), 2003854 (2021). 10.1002/aenm.202003854

