# Supplementary material for: Ultramicropore-Confined Solvation and Interphase Regulation Unlock High-Performance Hard Carbon Anodes for Sodium-Ion Batteries
Source: Nanomicro Lett. 2026 Jul 27;19:5. doi: 10.1007/s40820-026-02300-x (PMC13407412; doi:10.1007/s40820-026-02300-x)
Supplement: Supplementary file 1 — Supplementary file1 (DOCX 7467 KB) [file 40820_2026_2300_MOESM1_ESM.docx]

Supporting Information for

**Ultramicropore-Confined Solvation and Interphase Regulation Unlock High-Performance Hard Carbon Anodes for Sodium-Ion Batteries**

Shunyuan Tan^1^, Zhiyuan Cheng^1^, Jiahao Xing^1^, Jingkai Gao^1^, Zimo Huang^1,2^, Hongshuai Hou^3^, Zhongliang Tian^1,2^, Yanqing Lai^1,2^, Jie Li^1,2^, Simin Li^1,2^* and Xiaobo Ji^3^*

^1^ National Energy Metal Resources and New Materials Key Laboratory, School of Metallurgy and Environment, Central South University, Changsha 410083, P. R. China

^2^ National Engineering Research Center of Low-carbon Nonferrous Metallurgy, National Energy Metal Resources and New Materials Key Laboratory, Hunan Provincial Key Laboratory of Nonferrous Value-Added Metallurgy, Engineering Research Center of the Ministry of Education for Advanced Battery Materials, Changsha 410083, P. R. China

^3^ College of Chemistry and Chemical Engineering, National Energy Metal Resources and New Materials Key Laboratory, State Key Laboratory of Powder Metallurgy, Central South University, Changsha 410083, P. R. China

* Corresponding authors. E-mail: [simin.li@csu.edu.cn](mailto:simin.li@csu.edu.cn) (Simin Li); [xji@csu.edu.cn](mailto:xji@csu.edu.cn) (Xiaobo Ji)

**S1 Supporting Figures**


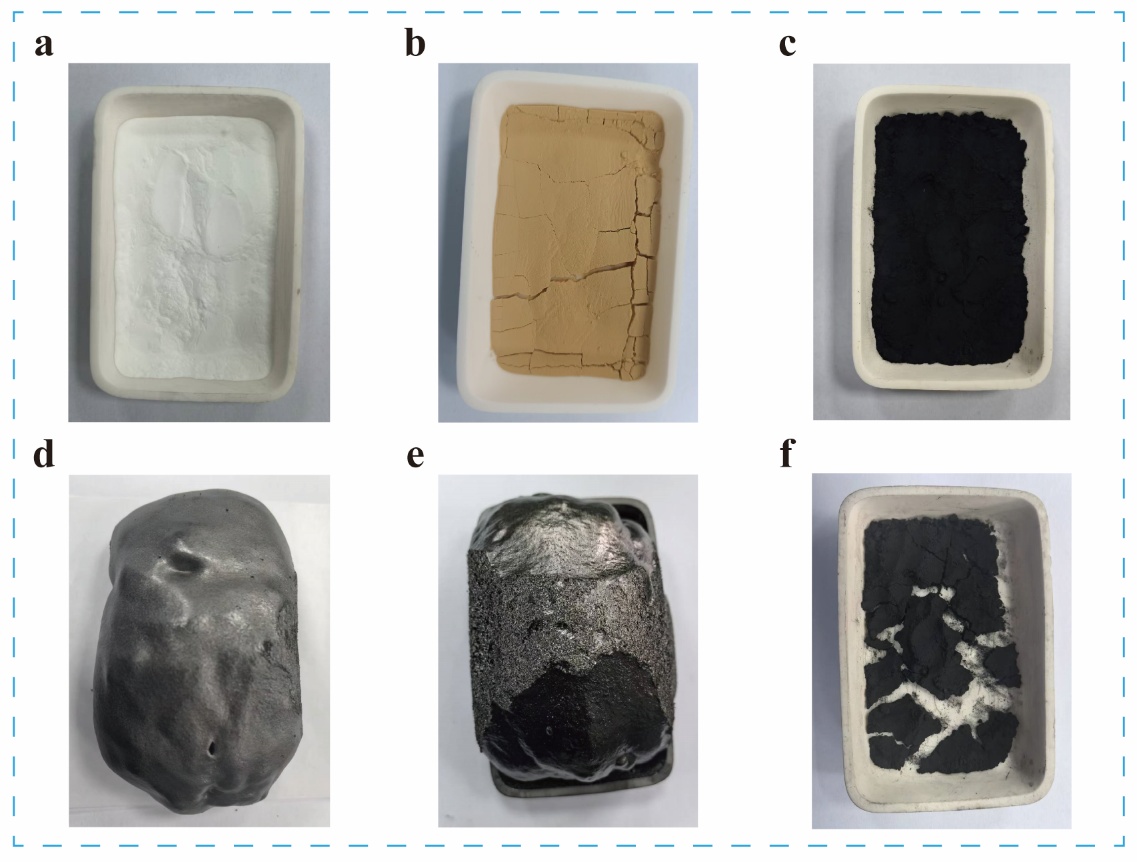


**Fig. S1** Optical photographs of **a** S, **b** OS, and **c** IOS, showcasing their morphological evolution after pyrolysis at 600 °C for 1 h in **d** S-600, **e** OS-600, and **f** IOS-600.





**Fig. S2** TG-DSC curve of S in air.

The thermogravimetric analysis-differential scanning calorimetry (TG-DSC) curve of S under an air atmosphere exhibits a small endothermic peak near 65 °C, corresponding to the evaporation of physically adsorbed water. A broad endothermic region appears between 120 and 290 °C, although no distinct peak is observed within this range. A pronounced exothermic peak is detected from 293 to 570 °C, indicating that thermal decomposition occurs concurrently with the melting of S microcrystals, meaning that decomposition begins prior to the complete melting of the crystalline domains.





**Fig. S3** TG curve of S, OS and IOS in Ar.


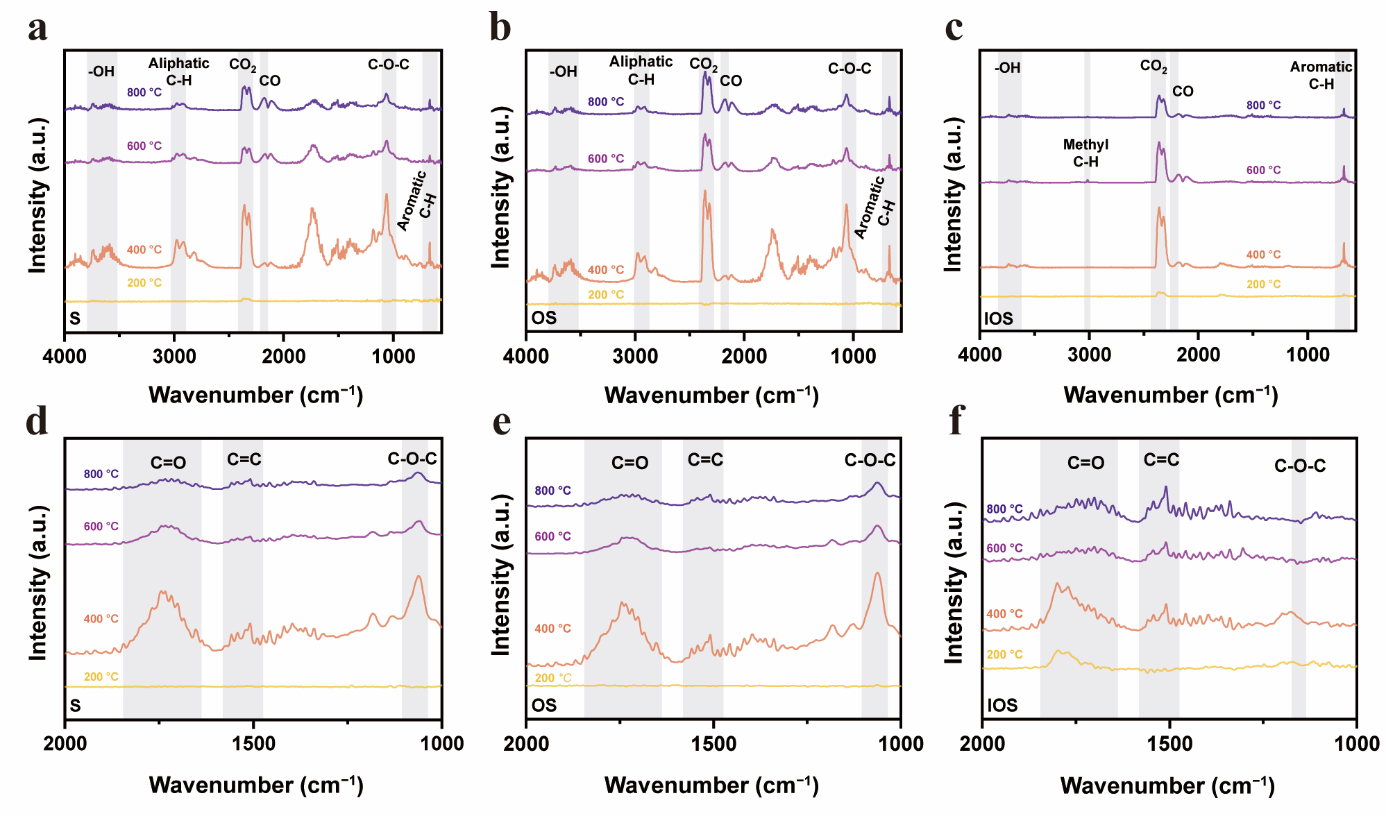


**Fig. S4** FTIR spectra and corresponding enlarged views of gases evolved from **a**, **d** S, **b**, **e** OS, and **c**, **f** IOS at different temperatures.


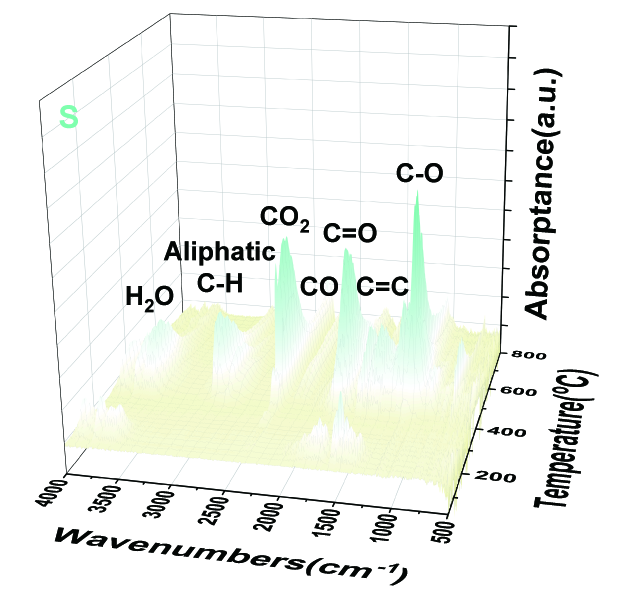


**Fig. S5** TG-FTIR analysis of gas evolution from S.


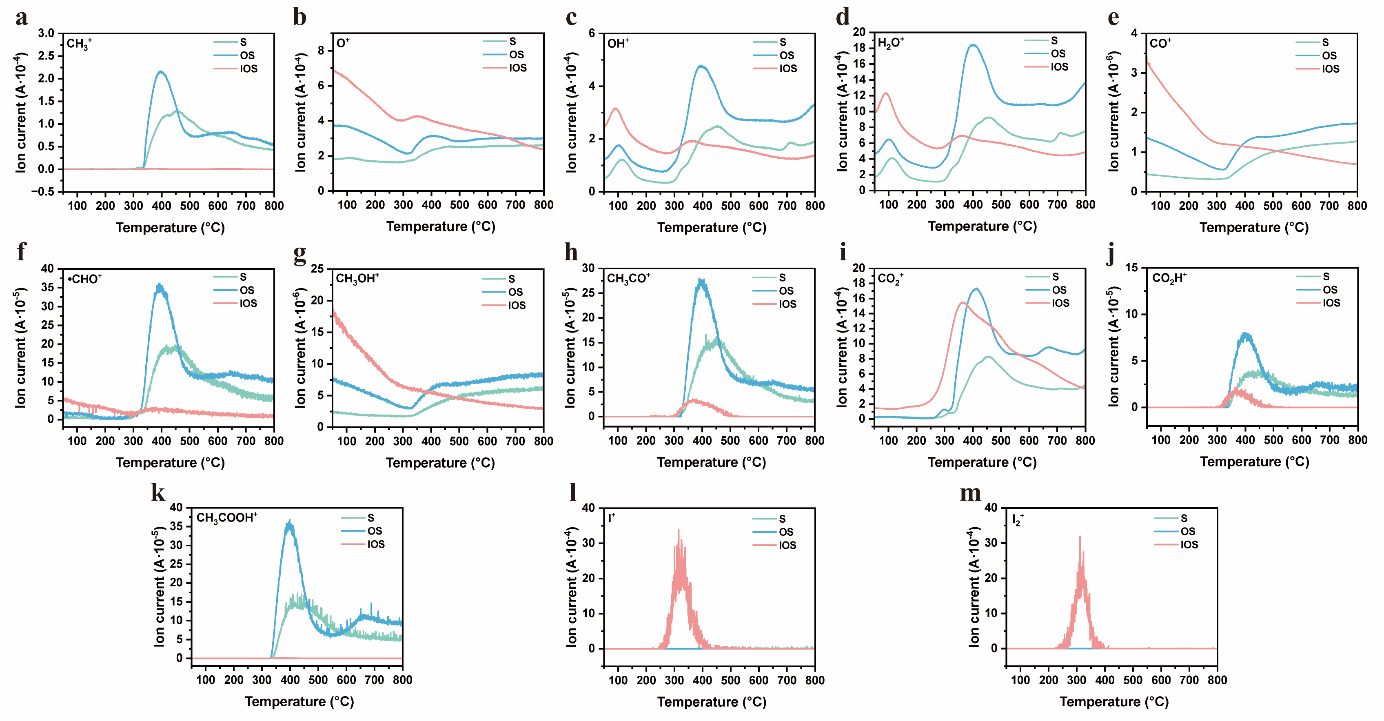


**Fig. S6** MS analysis of gases evolved during the pyrolysis of S, OS, and IOS under an Ar atmosphere. The major mass fragments are presented in descending order of intensity **a–m**: *m*/*z* 15 (CH_3_^+^), 16 (O^+^), 17 (OH^+^), 18 (H_2_O^+^), 28 (CO^+^), 29 (•CHO^+^), 32 (CH_2_OH^+^), 43 (CH_3_CO^+^), 44 (CO_2_^+^), 45 (CO_2_H^+^), 60 (CH_3_COOH^+^), 127 (I^+^), and 254 (I_2_^+^).

For S, pyrolysis is dominated by glycosidic bond cleavage and subsequent levoglucosan (LG) formation above ~280 °C. The volatilized LG further undergoes ring-opening fragmentation and secondary oxidation reactions, generating H_2_O, aldehyde fragments (*m/z* 29, •CHO^+^), acetyl fragments (*m/z* 43, CH_3_CO^+^), CO (*m/z* 28), and minor CO_2_ (*m/z* 44). Continuous evolution of these gaseous species disrupts the carbon backbone and promotes severe melt foaming during carbonization.

In OS, the pyrolysis chemistry is partially redirected by the pre-introduction of oxygenated functionalities during oxidative pretreatment. Oxygen-containing groups are incorporated primarily through hydrogen abstraction from hydroxyl groups, generating alkoxy intermediates that subsequently evolve into aldehyde and carboxyl functionalities [1]. Upon heating, aldehydes further transform into carboxylic acids (*m/z* 45, CO_2_H^+^), while adjacent carboxyl groups undergo dehydration condensation to form anhydride-related intermediates, as evidenced by the characteristic *m/z* 60 (CH_3_COOH^+^) signal. Based on these observations, the pyrolysis behavior of OS can be described by a “hydroxyl–aldehyde–carboxylic acid–anhydride” pathway. This process is further supported by the intensified MS signals at *m/z* 29, 43, 44, and 60. Formation of anhydride-like structures partially suppresses melt foaming by promoting dehydration and crosslinking reactions before extensive volatilization occurs [2]. However, owing to the relatively short oxidation duration (6 h), glycosidic bond cleavage and LG generation are not completely inhibited. Consequently, the anhydride-forming pathway still competes with LG-driven volatilization and fragmentation, resulting in residual foaming and incomplete framework stabilization in OS.


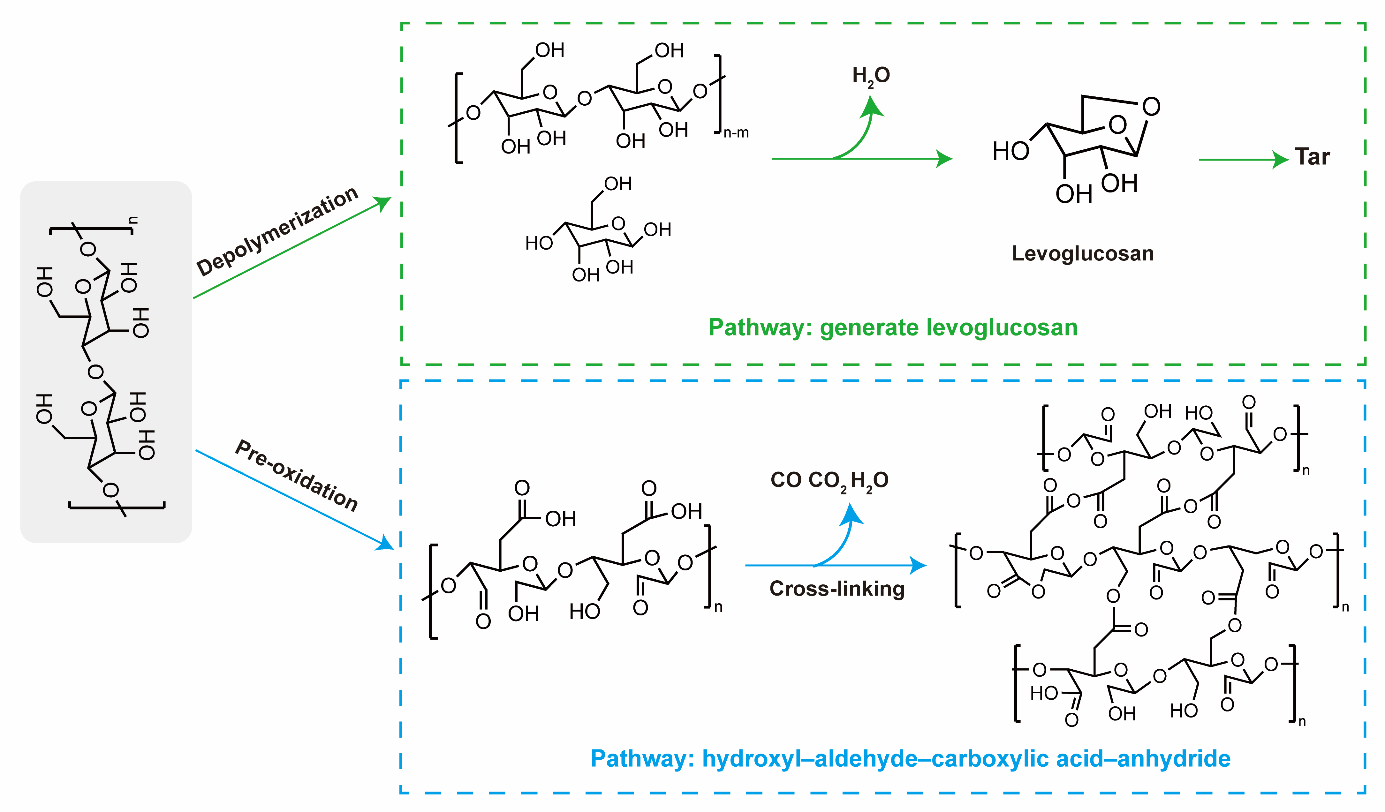


**Fig. S7** Proposed pyrolysis pathways of OS, illustrating the competitive formation of levoglucosan and the “hydroxyl–aldehyde–carboxylic acid–anhydride” pathway [S1–S3].


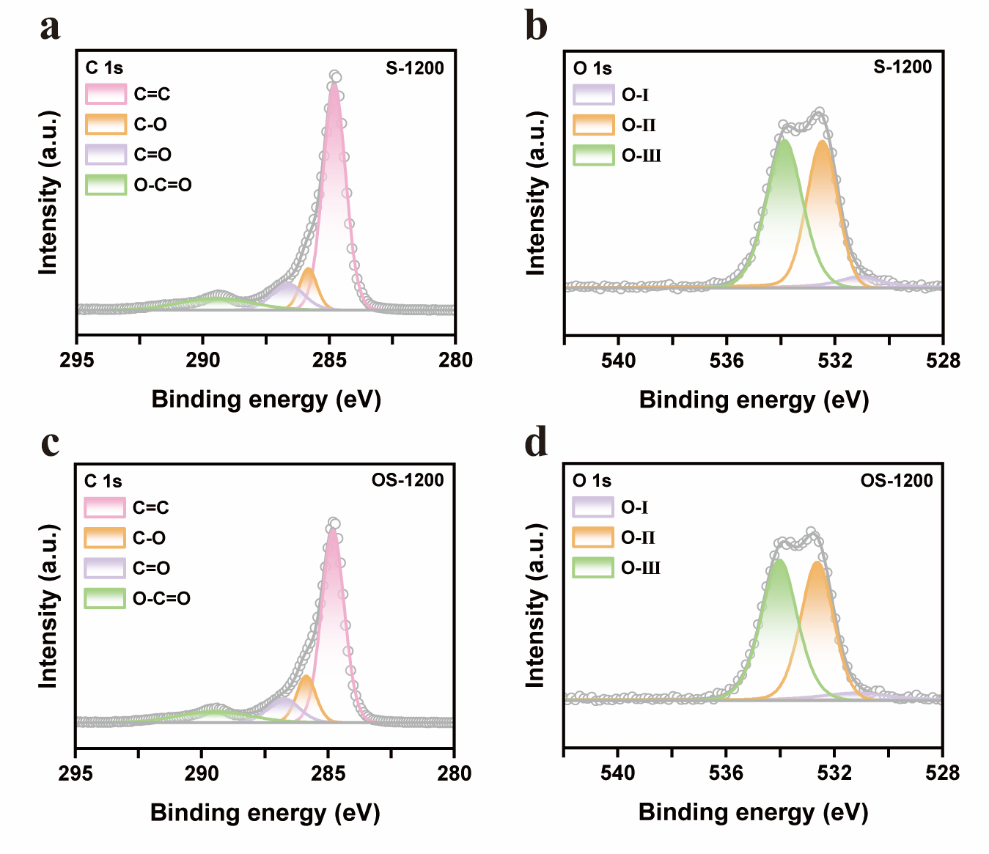


**Fig. S8** High-resolution XPS spectra of C 1s and O 1s for **a–b** S-1200 and **c–d** OS-1200.


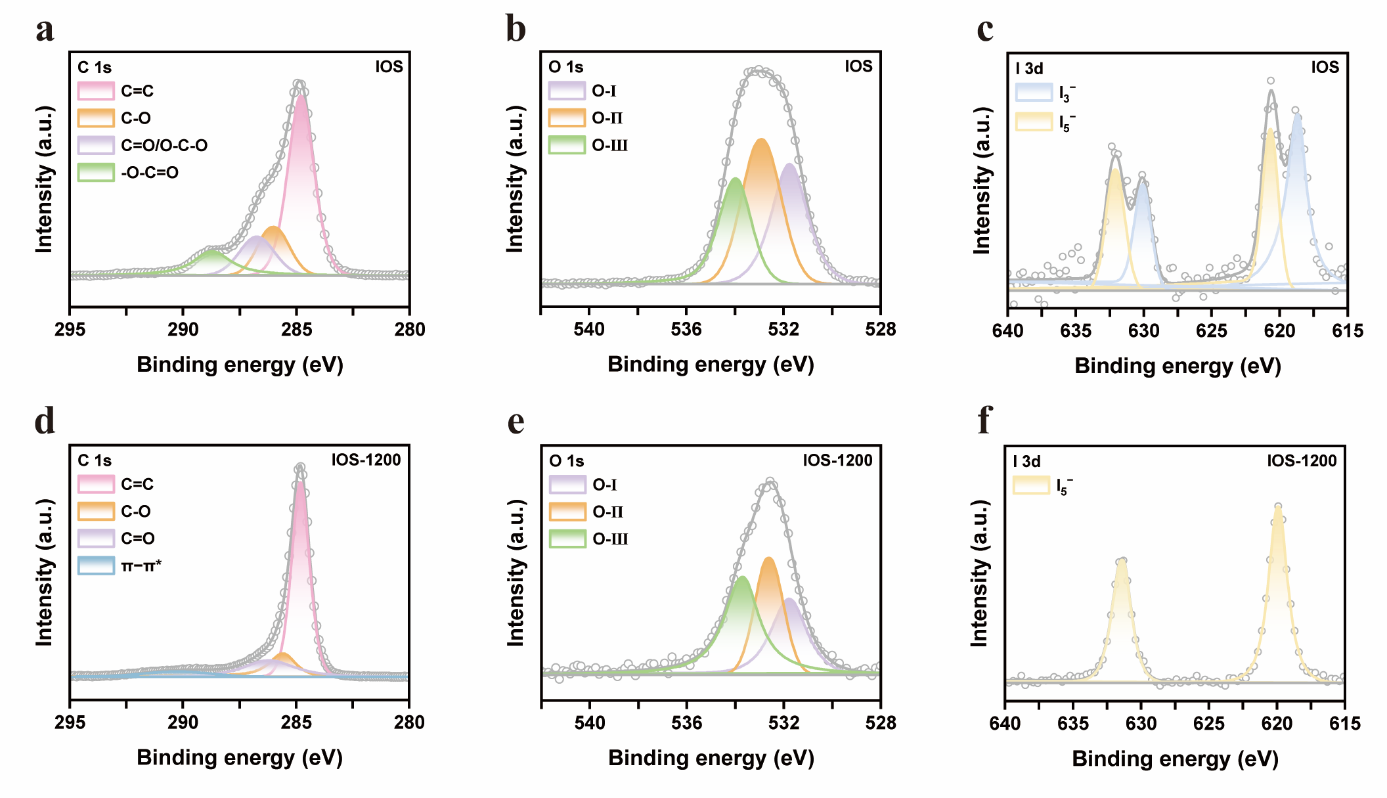


**Fig. S9** High-resolution XPS spectra of C 1s, O 1s, and I 3d for **a–c** IOS and **d–f** IOS-1200.

Figure S9c, f shows the I 3d spectra of IOS and IOS-1200. IOS contains coexisting I_3_^−^ and I_5_^−^ species, while IOS-1200 is dominated by I_5_^−^. The relative fractions of I_3_^−^ and I_5_^−^ are summarized in Table S1. These observations indicate a temperature-dependent redistribution of polyiodide species during carbonization, representing the residual iodine states in the final carbonized material.


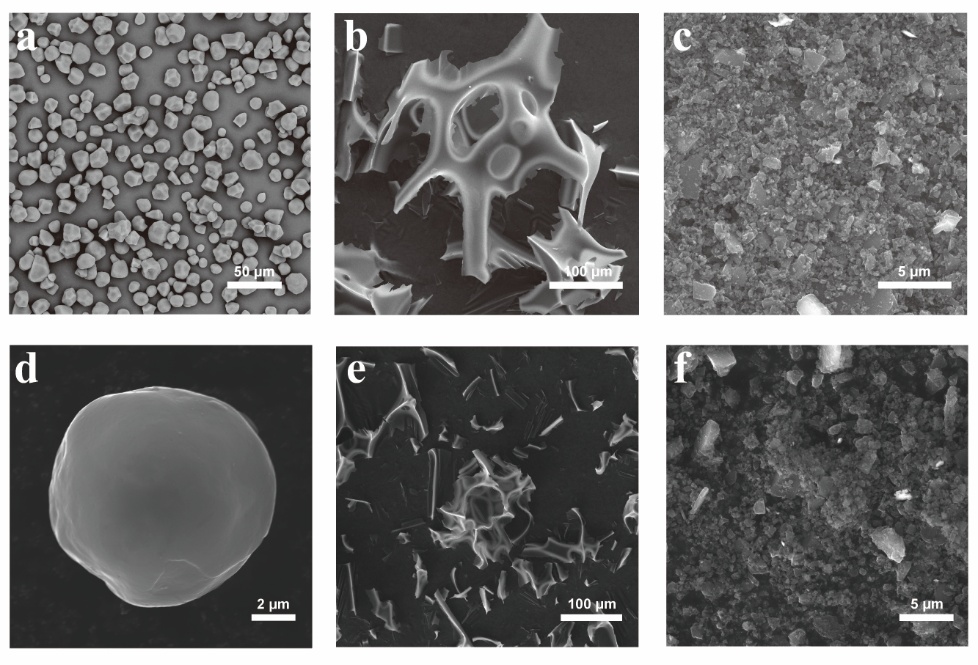


**Fig. S10** SEM images of **a** S, **b, c** S-600 before and after ball milling, respectively, **d** IOS-1200, and **e, f** OS-600 before and after ball milling, respectively.

The S consists of particles ranging in shape from polyhedral to nearly spherical (Fig. S10a), with diameters of approximately 5–25 µm. After pyrolysis, S-600 and OS-600 exhibit foam-like architectures (Fig. S1d, e), and their SEM images reveal thin-film morphologies (Fig. S10b, e), which transform into granular flakes upon ball milling (Fig. S10c, f). Carbonization at 1200 °C causes pronounced particle fusion and agglomeration in S-1200 and OS-1200 (Fig. 2a, b), indicating incomplete decomposition of the sugar-ring structures.


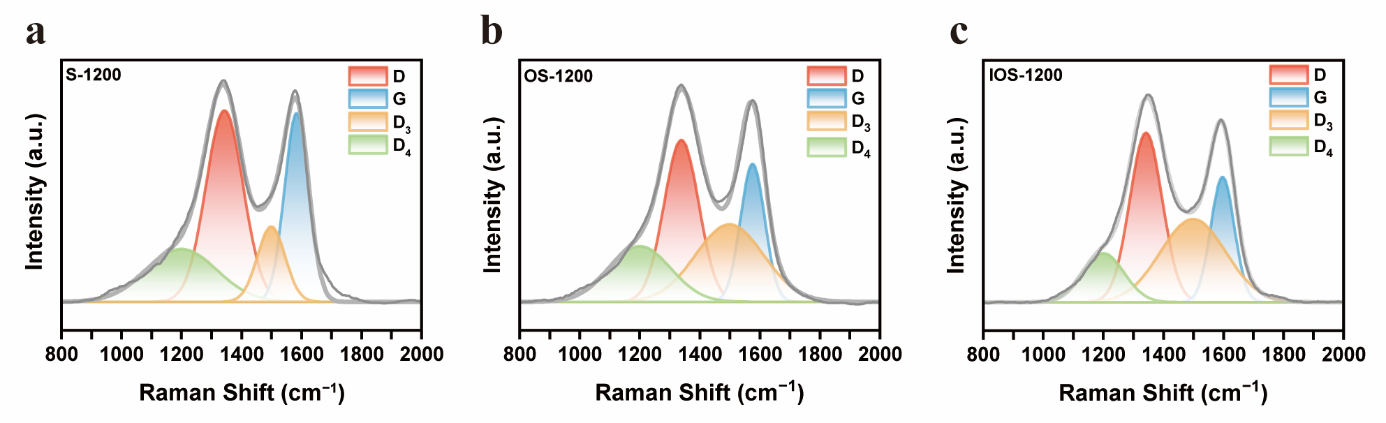


**Fig. S11** Raman spectra and fitted curves of S-1200, OS-1200 and IOS-1200.

The G band at 1580 cm^–1^ corresponds to the in-plane vibration of sp^2^-hybridized graphitic carbon with E_2_g symmetry. The D band at 1350 cm^–1^ is associated with disordered carbon present at graphite edges or structural defects with A_1_g symmetry. The D_3_ band at 1500 cm^–1^ originates from short-range sp^3^ carbon vibrations in amorphous regions, while the D_4_ band at 1200 cm^–1^ is linked to disordered graphitic lattices.





**Fig. S12** The fitted SAXS patterns of IOS-1200

The SAXS profiles were further analyzed using a sphere and power-law model in SasView to obtain additional information regarding the closed-pore structure of IOS-1200. The sphere component was employed to capture the nanoscale structural feature associated with closed ultramicropores, while the power-law contribution represents the pore–carbon interfacial scattering behavior.

The fitting results reveal a Porod exponent close to 4, suggesting relatively compact pore–carbon interfaces and dense carbon walls. Considering the highly disordered nature of HC, the fitted sphere contribution should be regarded as an equivalent structural correlation feature rather than an ideal geometric spherical pore model.





**Fig. S13** CV profiles of OS-1200 at a scan rate of 0.1 mV s^−1^.


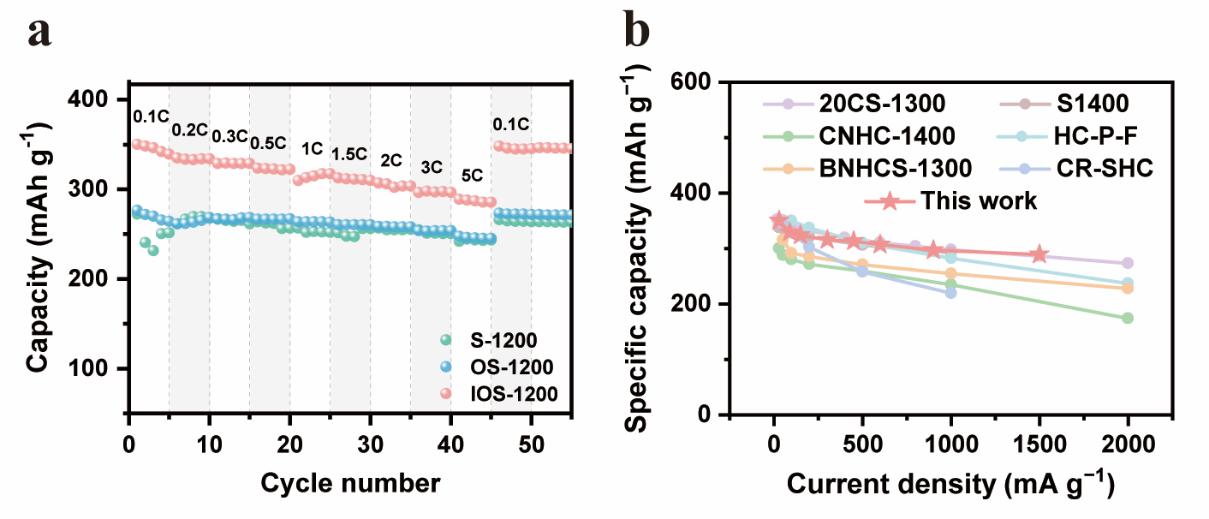


**Fig. S14.** **a** Rate capability of the three HC samples. **b** Comparative rate performance of IOS-1200 and previously reported carbon anodes [S4–S9].


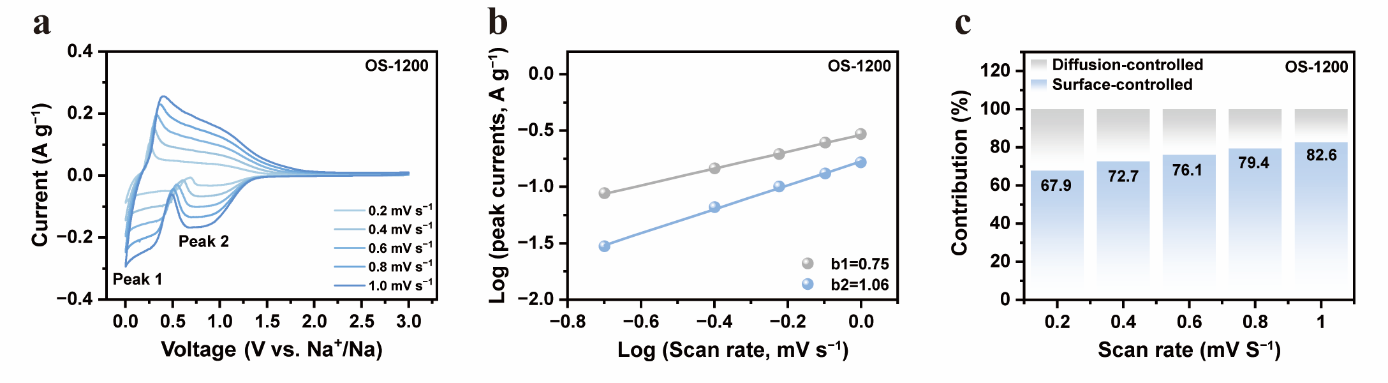


**Fig. S15** **a** CV curves at different scan rates, **b** linear fitting between log ($v$) and log ($i$) based on the power‑law relationship, and **c** quantitative capacitive contribution at various scan rates of OS‑1200.


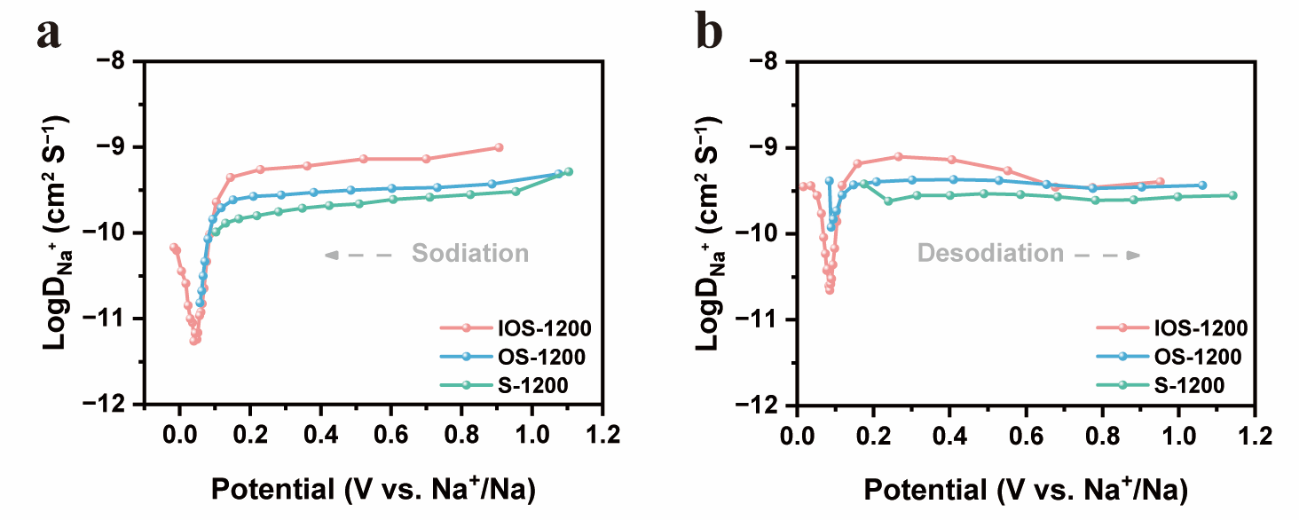


**Fig. S16** Na^+^ diffusion coefficients (*D*_Na⁺_) derived from GITT analysis during the **a** sodiation and **b** desodiation processes for S-1200, OS-1200, and IOS-1200.


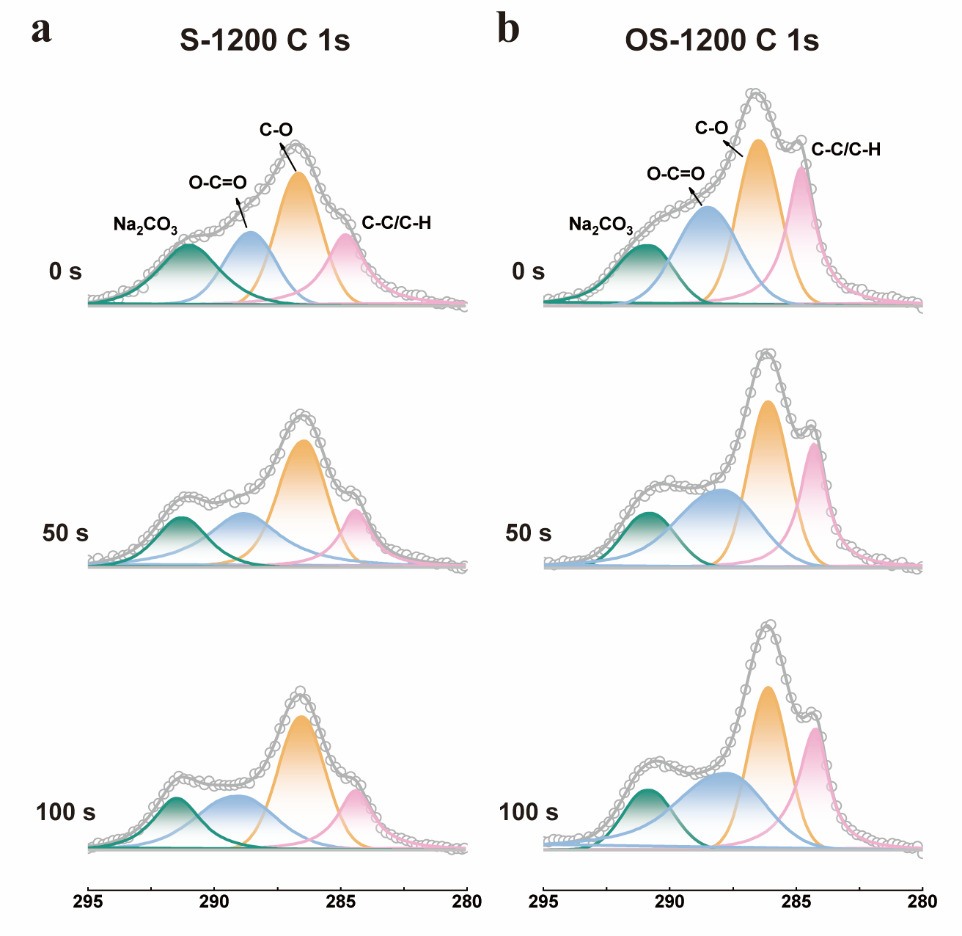


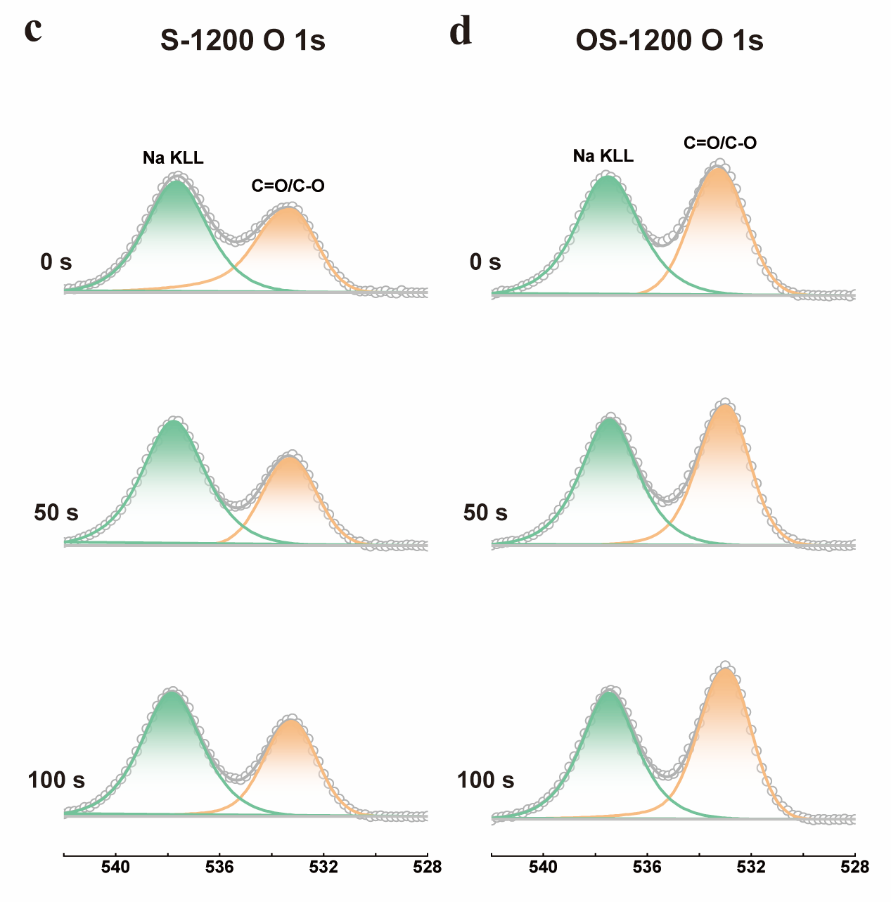


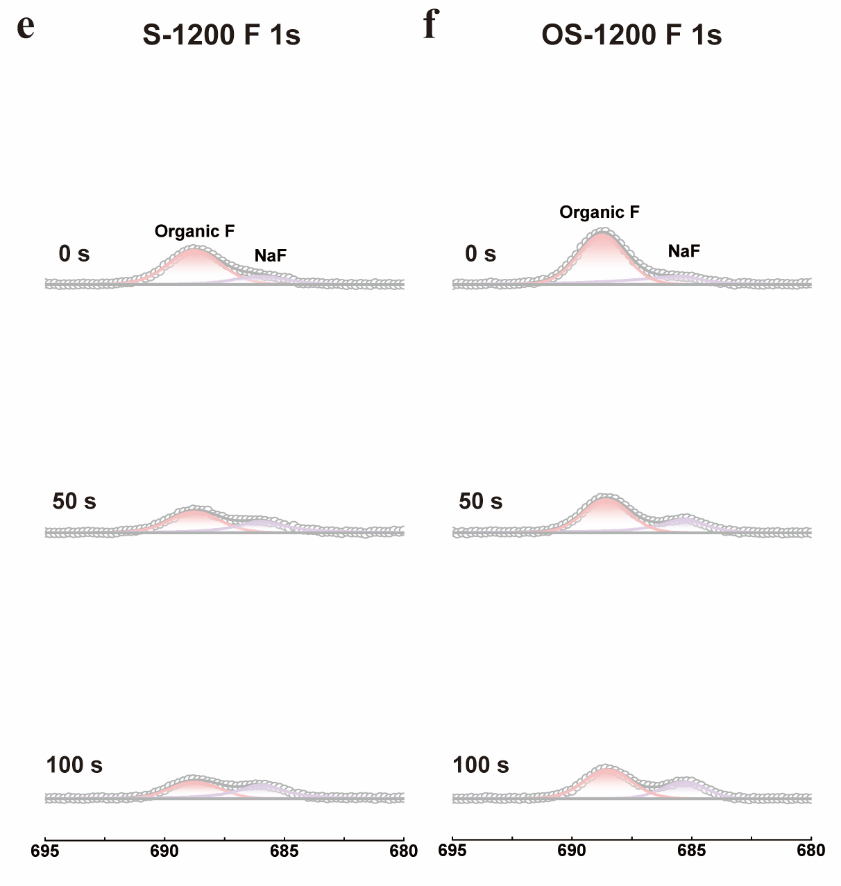


**Fig. S17** Depth-profiling XPS spectra of the SEI on the S-1200 and OS-1200 anode after 10 cycles charged to 3.0 V: **a–b** C 1s, **c–d** O 1s, and **e–f** F 1s.

Notably, the XPS spectra for S-1200 and OS-1200 (Fig. S17) are plotted on the same relative intensity scale (a.u.) as those for IOS-1200 (Fig. 4d–f) to facilitate direct comparison. Therefore, substantial blank spaces appear in Fig. S17e**–**f, as the F 1s peak intensities of S-1200 and OS-1200 are much lower relative to that of IOS-1200.


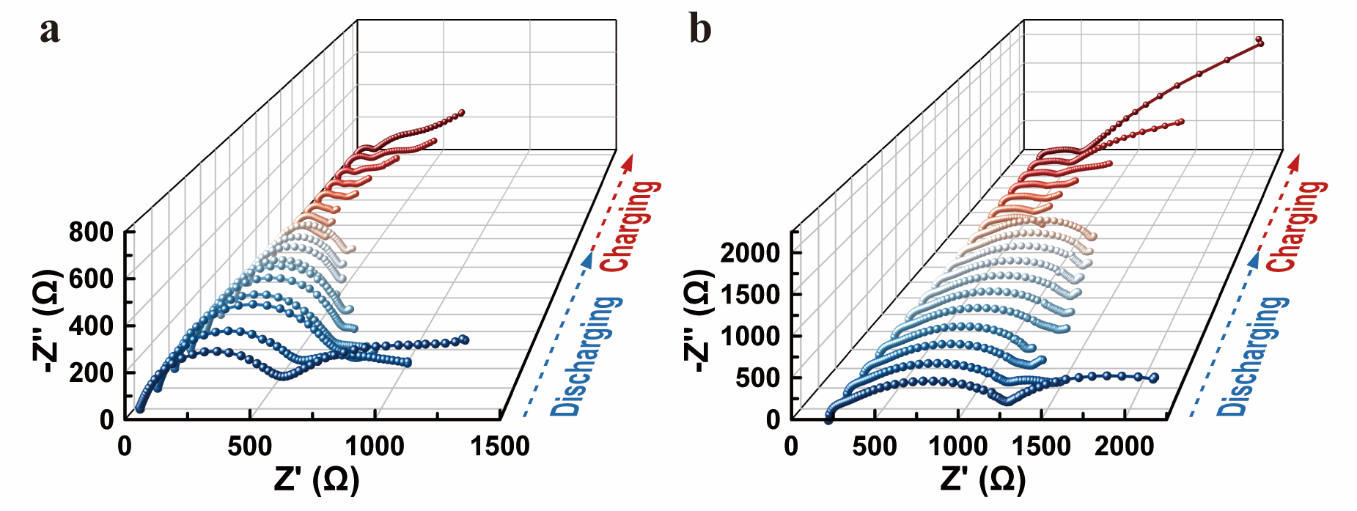


**Fig. S18** In situ EIS profiles recorded during the discharging and charging cycling of **a** IOS-1200 and **b** OS-1200.





**Fig. S19** DRT curve fitted from EIS spectra for OS-1200.


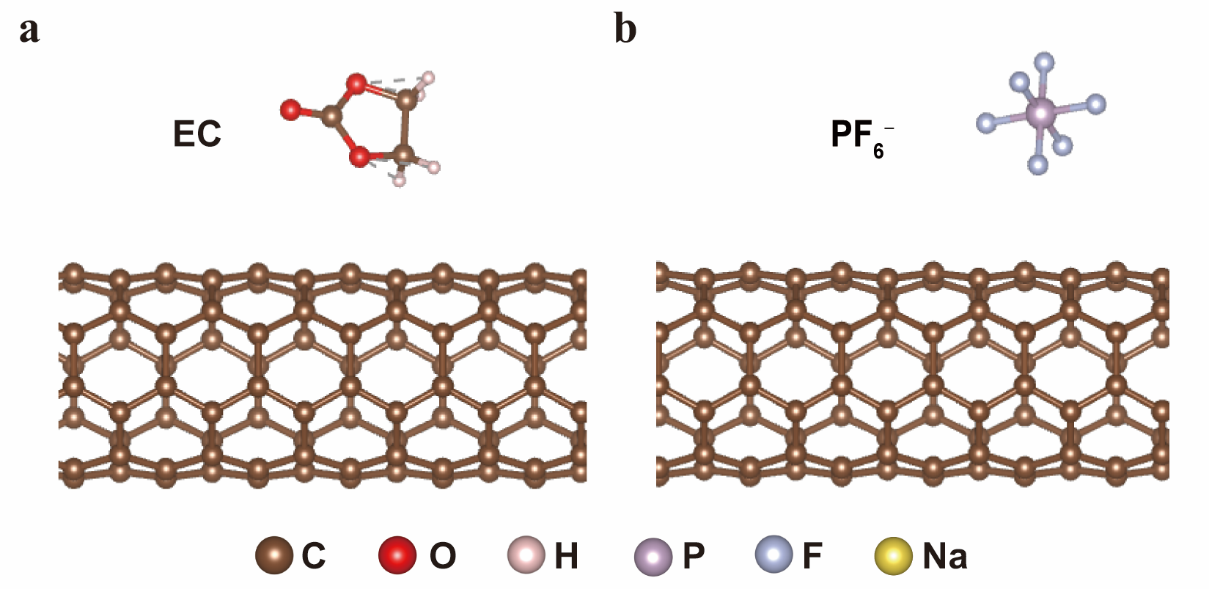


**Fig. S20** Schematic adsorption model of EC and PF_6_^−^ on the pure carbon surface.





**Fig. S21** Selective in situ XRD patterns of IOS-1200 during discharge and charge process.

**
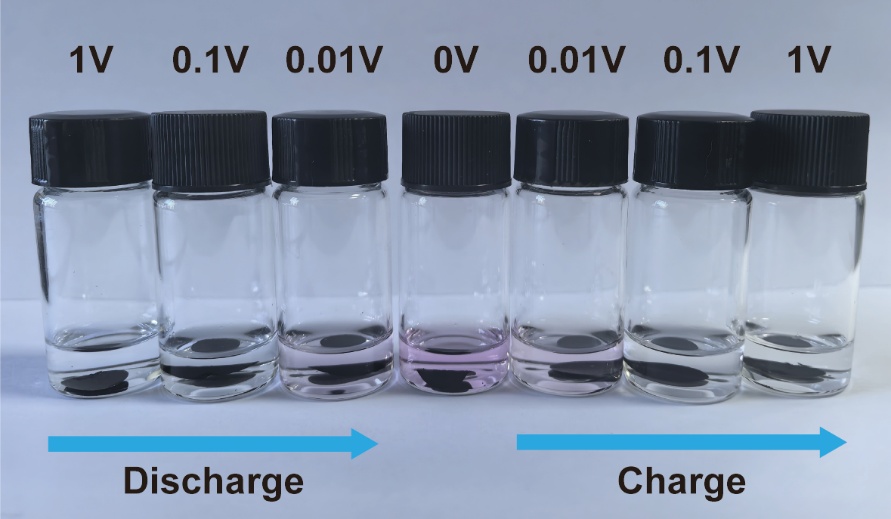
**

**Fig. S22** Picture of the reaction of IOS-1200 with ethanol solution containing 1% phenolphthalein at different potentials.

**S2 Supporting Tables**

**Table S1** Fitting atomic proportion of O 1s, C 1s and I 3d spectra in the XPS pattern of the different HC materials

| Sample | O 1s (%) | | |  | C 1s (%) | | | |  |  | I 3d (%) | |  |
| --- | --- | --- | --- | --- | --- | --- | --- | --- | --- | --- | --- | --- | --- |
|  | O-Ⅰ | O-Ⅱ | O-Ⅲ |  | C=C | C–O | C=O | O-C=O | π–π* |  | I_3_^−^ | I_5_^−^ | |
| S-1200 | 0.47 | 3.05 | 3.50 |  | 62.91 | 8.90 | 10.44 | 10.73 | / |  | / | / | |
| OS-1200 | 0.50 | 3.48 | 3.88 |  | 58.92 | 12.19 | 10.14 | 10.89 | / |  | / | / | |
| IOS-1200 | 1.71 | 1.75 | 2.45 |  | 59.10 | 13.22 | 15.25 | / | 5.91 |  | / | 0.62 | |
| IOS | 8.03 | 9.88 | 6.65 |  | 40.34 | 12.7 | 11.47 | 10.55 | / |  | 0.22 | 0.16 | |

**Table S2** The first cycle discharge/charge capacity and ICE

| Sample | Discharging capacity  (mAh g^-1^) | Charging capacity  (mAh g^-1^) | ICE  (%) |
| --- | --- | --- | --- |
| S-1200 | 394.63 | 287.63 | 72.96 |
| OS-1200 | 385.79 | 288.59 | 74.81 |
| IOS-1200 | 399.10 | 352.85 | 88.41 |

**Supplementary References**

1. J. Huang, E. Li, B. Dai, et al., Regulating the active hydroxyl group of starch: revealing the evolution of hard carbon structure and sodium storage behavior. Carbon **229**, 119527 (2024). <https://doi.org/10.1016/j.carbon.2024.119527>
2. J. Huang, S. Liu, E. Li, et al., Double functionalization strategy: using acetate metal salt as medium to optimize hard carbon. Carbon **234**, 119981 (2025). <https://doi.org/10.1016/j.carbon.2024.119981>
3. S. Jia, B. Zhang, J. Gao, et al., Biomass-derived hard carbon anodes: From structural engineering to industrial sodium-ion battery applications. Energy Storage Mater. **80**, 104420 (2025). <https://doi.org/10.1016/j.ensm.2025.104420>
4. H. Zhong, Q. Huang, M. Zou, et al., From food to hard carbon: citric acid enhanced biomass-derived anodes for high-performance sodium storage. Chem. Eng. J. **508**, 160879 (2025). <https://doi.org/10.1016/j.cej.2025.160879>
5. S. Xu, W. Liu, S. Mao, et al., Lotus root starch derived sustainable hard carbon for fast-charging sodium-ion batteries. Chem. Eng. J. **519**, 165014 (2025). <https://doi.org/10.1016/j.cej.2025.165014>
6. H. Zeng, J. Zhang, J. He, et al., Starch-derived N-doped hard carbons for sodium-ion storage: preparation and enhanced electrochemical performance. J. Power Sources **654**, 237850 (2025). <https://doi.org/10.1016/j.jpowsour.2025.237850>
7. G. Yang, J. Zhang, Z. Zhang, et al., Surface functionalized porous spherical hard carbon material derived from taro starch for high performance sodium storage. Electrochim. Acta **521**, 145935 (2025). <https://doi.org/10.1016/j.electacta.2025.145935>
8. X. Ma, J. Hu, W. Zhang, et al., An effective B-doped strategy to regulate the closed-pore formation of hard carbon nanospheres for sodium-ion batteries. J. Energy Storage **126**, 117149 (2025). <https://doi.org/10.1016/j.est.2025.117149>
9. C. Yu, Y. Guan, P. Huang, et al., Crosslinked starch-derived hard carbon with abundant closed pores for enhanced plateau capacity in sodium-ion storage. Carbon **247**, 121020 (2026). <https://doi.org/10.1016/j.carbon.2025.121020>
